# On Deep Landscape Exploration of COVID-19 Patients Cells and Severity Markers

**DOI:** 10.3389/fimmu.2021.705646

**Published:** 2021-09-16

**Authors:** Aarón Vázquez-Jiménez, Ugo Enrique Avila-Ponce De León, Meztli Matadamas-Guzman, Erick Andrés Muciño-Olmos, Yoscelina E. Martínez-López, Thelma Escobedo-Tapia, Osbaldo Resendis-Antonio

**Affiliations:** ^1^Human Systems Biology Laboratory, Instituto Nacional de Medicina Genómica (INMEGEN), Mexico City, Mexico; ^2^Programa de Doctorado en Ciencias Biológicas, UNAM, Mexico City, Mexico; ^3^Programa de Doctorado en Ciencias Biomédicas, UNAM, Mexico City, Mexico; ^4^Programa de Doctorado en Ciencias Médicas y de la Salud, UNAM, Mexico City, Mexico; ^5^Programa de Maestría y Doctorado en Ciencias Bioquímicas, UNAM, Mexico City, Mexico; ^6^Coordinación de la Investigación Científica - Red de Apoyo a la Investigación, UNAM, Mexico City, Mexico

**Keywords:** COVID-19, immune landscape, machine-learning, cell heterogeneity, immune system, single-cell analysis

## Abstract

COVID-19 is a disease with a spectrum of clinical responses ranging from moderate to critical. To study and control its effects, a large number of researchers are focused on two substantial aims. On the one hand, the discovery of diverse biomarkers to classify and potentially anticipate the disease severity of patients. These biomarkers could serve as a medical criterion to prioritize attention to those patients with higher prone to severe responses. On the other hand, understanding how the immune system orchestrates its responses in this spectrum of disease severities is a fundamental issue required to design new and optimized therapeutic strategies. In this work, using single-cell RNAseq of bronchoalveolar lavage fluid of nine patients with COVID-19 and three healthy controls, we contribute to both aspects. First, we presented computational supervised machine-learning models with high accuracy in classifying the disease severity (moderate and severe) in patients with COVID-19 starting from single-cell data from bronchoalveolar lavage fluid. Second, we identified regulatory mechanisms from the heterogeneous cell populations in the lungs microenvironment that correlated with different clinical responses. Given the results, patients with moderate COVID-19 symptoms showed an activation/inactivation profile for their analyzed cells leading to a sequential and innocuous immune response. In comparison, severe patients might be promoting cytotoxic and pro-inflammatory responses in a systemic fashion involving epithelial and immune cells without the possibility to develop viral clearance and immune memory. Consequently, we present an in-depth landscape analysis of how transcriptional factors and pathways from these heterogeneous populations can regulate their expression to promote or restrain an effective immune response directly linked to the patients prognosis.

## 1 Introduction

COVID-19 derives from SARS-CoV-2 infection, having diverse clinical symptoms according to the infection severity. There are still many unresolved questions regarding the disease pathogenesis and the reasons underlying the high variations on clinical courses, ranging from asymptomatic forms to severe manifestations. So far, illness severity is cataloged according to the clinical manifestations. However, physiological symptoms do not always reflect the capability of patients to overcome the disease. A molecular characterization based on signatures and alterations at the cellular level according to illness severity can streamline the clinical management and treatment. Interestingly, the responses of immune and non-immune cells recruited during the infection determine the clinical outcome of the disease. Recent single-cell studies show the differences of these cells, characterizing the immune landscape during infection (1–5). Through various cytokines and chemokines, the interaction between these cells is essential to determine the clinical outcome. Clinical symptoms associated with SARS-CoV-2 comprise several dominant processes linked to tissue inflammation, usually given by the cytokine storm (6–9). The cytokine storm is an uncontrolled over-production of soluble inflammation markers that sustain an aberrant systemic inflammatory response (10, 11). During COVID-19, pro-inflammatory cytokines such as interferon, interleukins (IL-6, IL-12, IL-7), and chemokines (CXCL10 and CCL2) are essential to rule clinical complications (1, 10, 12–14). Neutrophils, T cells, B cells, and macrophages participate actively in the massive production of pro-inflammatory molecules (1, 12, 15). Despite these findings, the underlying mechanisms regulating this cytokine storm are unknown. The characterization of the molecular regulation of cell responses during the infection serves to understand fundamental mechanisms and potentially restructure the therapeutic schemes and treatments against COVID-19.

Recent single-cell data from bronchoalveolar lavage fluid (BALF) of 3 healthy controls and 9 COVID-19 patients described the cellular composition of immune cells and the expression of some cytokine/chemokine and chemokine receptors in each clinical response (1). Using this dataset, we identified possible biomarkers and scrutinized the potential mechanisms that characterize different disease severities. In this work, through a supervised machine-learning technique applied over the single-cell RNAseq data, we propose a computational model capable of classifying and distinguishing with high accuracy cells coming from COVID-19 patients with a moderate or severe response according to their genetic profiles. Besides, we suggest a potential genetic signature to classify moderate or severe response in patients with COVID-19. Furthermore, the output of this classifier can prioritize the level of attention of new patients based on their gene expression profiles using a BALF sample. Finally, to identify some molecular mechanisms underlying immune response on COVID-19 infection, we analyzed the diversity of cellular composition of some cells in the immunological system. Thus, we described the undergoing changes for every cell and how these changes promote COVID-19 severity altogether among patients. We suggest that severe patients had the innate and adaptive impaired immune response; innate cells activated the IL-6 pathway in a positive loop affecting antigen presentation that impacts adaptive response and immune cells maturation. Although this group seems to activate IFNs pathways, their activation might not be in the correct order to be effective. Overall, our study contributes to identifying potential disease severity biomarkers from BALF samples and dissecting the regulatory mechanisms and pathways potentially responsible for triggering an inadequate immunological response in patients with COVID-19.

## 2 Methods

### 2.1 Data

Raw scRNA-seq data of Covid-19 patients were obtained from GEO (GSE145926). To sum up, bronchoalveolar lavage fluid (BALF) cells were collected from 3 healthy controls and 9 patients grouped according to their symptoms as moderate, and severe. Samples were sequenced using 10x Genomics technology (1). Originally, there was one patient with critical symptoms. However, it was considered among the severe group due to the poor representation of the critical symptomatology.

### 2.2 Data Processing

The count matrix was filtered to exclude cells with <200 genes and mitochondrial gene percentage <0.05, we got 90696 cells within 25627 genes each one. Using the “seurat” v3.1.5 R package (16), data were log-normalized using the function ‘LogNormalize’ with the default parameters. To assess cell heterogeneity, we considered the 2000 genes with the highest variability using the ‘FindVariableFeatures’ function with the ‘vst’ method. Data were integrated into one object for all conditions (healthy, moderate, and severe). We applied a linear transformation to the data prior to the PCA computation. Dimensionality reduction was performed in the uMAP space using the top 20 principal components of the PCA. To perform data clustering, we used the Seurat graph-based approach with a resolution of 0.5.

### 2.3 Machine-Learning Analysis

As part of contributing to markers in moderate and severe stages of COVID-19 disease, we performed a machine-learning analysis on the scRNAseq data. To identify the set of genes that serve as biomarkers to differentiate the outcome of moderate and severe COVID-19 patients, we applied the Extreme Gradient Boosting (XGBoost) algorithm. This is a supervised algorithm to accomplish tree classification widely used in machine-learning (17, 18). All calculations were done in python (version 3.7.9) through the open source software library XGBoost (17). To this end, we proceeded as follows. Cells from data count matrices comprising moderate and severe patients were labelled as 0 and 1 respectively. Then, we randomly selected 75% of the entire samples to define the train set of the model, the rest of the data was used to integrate the test data. The XGBoost model was built with the train data set and evaluated its performance with the test data set. We applied a K-fold (n_split =5) cross-validation method to ensure the independence of results onto the way to split the training and test data. This step essentially consisted in dividing the entire data into five equal proportions. Then, we trained the model by selecting four proportions as training set and the rest as test dataset. To evaluate the performance of the model, we calculated the confusion matrix over the test datasets. We repeated this procedure five times, in each realization we selected a different proportion as the test data, and the rest as the training data set such that all possible combinations were taken into account. We evaluated in each realization the performance of the model and its dependence on the split of the data through the area under the curve (AUC) of ROC curves, see supplementary material. A graphical description of the entire pipeline is shown in **Figure S1**. XGBoost classifiers that sustain the **Figures 2A**, **2D** in main text are reported in the machine learning section at https://github.com/resendislab/Covid-19_scRNAseq. It was accomplished by selecting the following parameters: maxima depth 4, eta = 0.2, metric to evaluate AUC, and objective to evaluate binary:hinge. Iteration of the method was done through 1000 steps. We evaluated the relevance assessment of characterized genes through the shap-values (SHapley Additive exPlanations) for the XGBoost algorithm. Shap values utilizes a game-theoretical approach to proceed with the best interpretation and explanation of the output of our machine-learning model. To visually identify the most relevant genes that contribute to the classification, we plotted the aggregate explanation plot, which comprises the SHAP values for each gene and their average expression levels (19). The model without quality associated genes was obtained in the same way as described before, see **Figure S1**. Assessment of both models (with and without quality associated genes) was accomplished by utilizing recent and entirely independent scRNAseq data of BALF samples for 3 moderate and 6 severe patients (19).

### 2.4 Cell Type Annotation

To identify the cell type in each cluster, differentially expressed genes were gathered using the FindMarkers function and compared to marker genes for each cell type. We got the marker genes from two sources: 1) The LM22 compilation containing 22 functionally defined human immune subsets profiled by microarrays (20); 2) based on a study of the changes in gene expression in cell types involved in idiopathic pulmonary fibrosis (20). Cell types and their marker genes are described in **Table S1**. Therefore, we identified the differentially expressed genes using the Wilcoxon Rank Sum test using a p-value <0.01 threshold. Then, we computed cell proportions quantifying the number of cells in each cell type divided by the total cells for the respective healthy control or patient. We split the T & NK group into T cells, NK, and Neutrophils by data reintegration. Then, taking only this group, we normalized it, found the 2000 most variable genes, computed the first 30 principal components to run the uMAP, and the top 20 principal components to perform the clustering with a resolution parameter of 0.6.

Accordingly with the T & NK separation and using the same parameters, we analyzed the heterogeneity within epithelial cells. We used 14 marker genes to identify five epithelial subtypes: alveolar type I (AT1), alveolar type II (AT2), secretory, squamous, and ciliated (21, 22) (**Table S2**).

### 2.5 Data Re-Integration

Data from every cell type for groups control, moderate, and severe were reintegrated and re-clustered (as described above) using the top 20 principal components of the PCA and a resolution of 0.5. Data subsets representing each cell type were used to study their regulatory network inferences.

### 2.6 Responsive Genes and Regulatory Network Inference

Functional pathway analysis was performed with PROGENy by the computation of top 500 pathways activity scores (23, 24) implemented in the “progeny” v1.12.0 R package. To infer the transcription factors (TFs) based on single cell expression data we used DoRothEA (24, 25) implemented in the R packages “dorothea” v1.2.0 and “viper” v1.24.0. Dorothea is a comprehensive resource containing a curated collection of transcriptional factors that compute their activity from the variations of the mRNA levels of their transcriptional targets. The activity is calculated *via* Viper which obtains a p-value and a normal enrichment score when it compares the regulon enrichments score with a null model which is randomly generated by permuting the samples. We took the top TFs value for each population: 50 for macrophages, monocytes and epithelial cells, 30 for T/NK cells and 100 for dendritic and B cells. A differentially expressed analysis and clustering were performed based on the TF activity, the used parameters were: the top 50 principal components of the PCA, a resolution equal to 0.8, uwot method, and the cosine metric. We used the framework provided by Seurat.

### 2.7 Pathway Enrichment Analysis

Pathway enrichment analysis was done using the Gene Set Enrichment Analysis (GSEA) (26). Gene sets were obtained from the MsigDB database, we used the hallmarks (27) and curated (C2) datasets. Statistical significance was assigned with an FDR < 0.05 and p-value < 0.01. In the specific case of dendritic cells, we performed a GSEA using the “webgestalt” R package v.0.4.4 and the TFs activity values from DoRothEA as input, and for the enrichment analysis we utilized KEGG, Gene Ontology-Biological Process (GO-BP), Wikipathway, Panther, and Reactome databases. Statistical significance was assigned with an FDR < 0.05. The parameters sigMethod, minNum, reportNum and perNum were changed from the default values to top, 5, 30 and 10000, respectively.

## 3 Results

### 3.1 Different Cell Populations Among COVID-19 Patients

**Figure 1:**
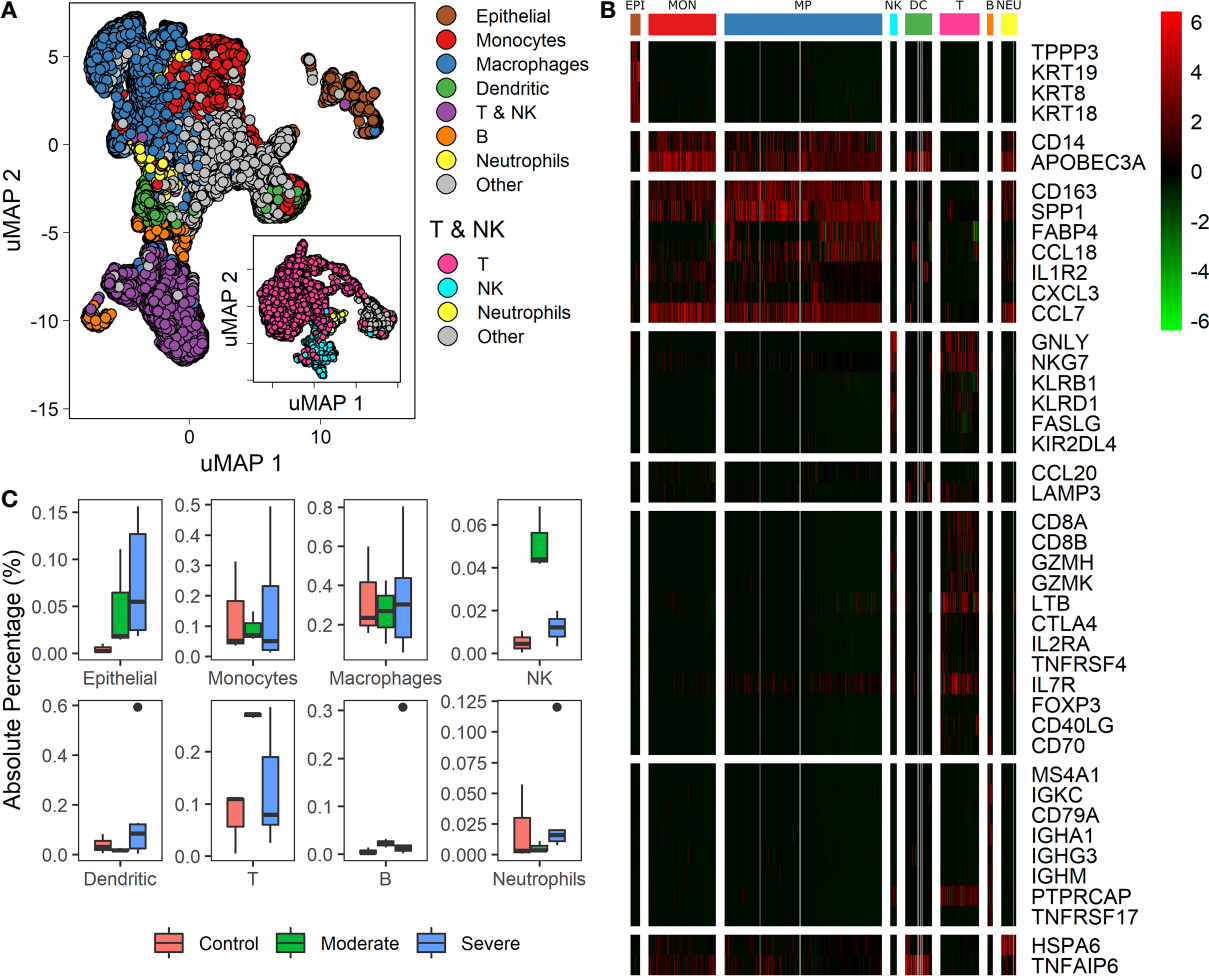
Differences in cell types among patients with diverse infection severity. **(A)** Umap projection of single cell data showing diverse cell types identified based on biomarkers (**Table S1**). The inset shows the uMAP projection and separation for the T & NK group. **(B)** Heatmap showing differentially expressed genes of clusters (columns) used to identify the cell types (rows): EPI, Epithelial cells; MON, monocytes; MP, macrophages; NK, natural killers cells; DC, dendritic cells; T, T cells; B, B cells; NEU, neutrophils. Rows dividers are related to each cell type, and the column groups set the cluster associated to each cell type. Colorbar shows the genes normalized expression values. **(C)** Boxplot for every cell-type identified, proportions of each cell type among healthy controls, moderate and severe patients.

Recently single-cell RNA studies on patients with COVID 19 suggest that specific immune and non-immune cells determine the disease severity (1, 3, 9, 28). We used data from bronchoalveolar lavage fluid (BALF) cells from 9 patients and 3 healthy controls. Patients were grouped according to the severity of the COVID-19 infection as moderate, and severe (1, 3). We mapped single-cell expression data on a Uniform Manifold Approximation and Projection (uMAP) to identify cell subpopulations correlated with the infection severity. Clustering analysis over the samples showed 22 clusters with different gene expression profiles (**Figure S2**). To ensure a proper cell type identification across data, we used expression profiles described in the LM22 immune compilation and idiopathic pulmonary fibrosis (23, 24). A total of 43 genes classified the data into 10 subpopulations: epithelial, monocytes, macrophages, dendritic, T, natural killer (NK), B, neutrophils, and “other” (**Figures 1A, B**, and **Table S1**). We identify T and NK cells in two steps: First, we separate both groups of cells as one named T & NK (**Figure 1A**). Second, taking the cells of the T & NK group, we reanalyzed data to separate subpopulation, under this reanalysis we identified T cells, NK, neutrophils and others (**Figure 1A**: inset). Group named as “other” gathered cells that we were unable to associate with a specific phenotype. These cell-types correspond to cells typically found in BALF (1, 3, 9, 29). We found differences in cell-types proportions compared to other reports using the same database (1, 15). These differences emerged because we used more gene markers to increase the selectivity of cell subpopulations and changes in some pipeline parameters. Besides, we determined the proportion of each cell type among healthy controls and patients (**Figure 1C**). Box-plots represent absolute percentage data distribution within patients and healthy controls for every cell type. At a glance, moderate patients showed an increased proportion of Epithelial, NK, T and B cells than healthy controls. Additionally, severe patients showed a higher dispersion of all cells-types. Although the proportion of macrophages does not change for patients and healthy controls, different studies have confirmed the change in the proportions for the other cell-types (3, 5, 10, 12). These findings are remarkable to find associations with clinical disease responses. However, we should be cautious on the interpretation as cell-types populations change dramatically over the course of active viral infections, given that these proportions are affected by clinical and genetic factors, sampling dates, and the number of days since the onset of symptoms (1, 3, 9, 29). Heterogeneity in cell composition supplies valuable information to survey cellular differences among patients with distinct responses to infection. As a consequence, we wondered if we could find a set of genes useful to classify the disease severity among patients using this dataset.

### 3.2 Cells From COVID-19 Patients Are Accurately Classified Through a Specific Gene Signature

**Figure 2:**
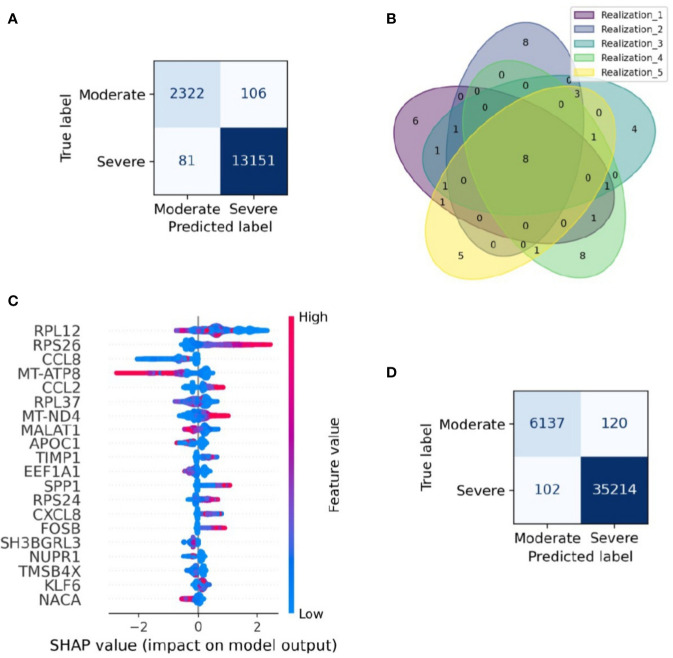
Gene signature able to classify single-cell data from moderate and severe patients. Gene signature able to classify single-cell data from moderate and severe patients. **(A)** Confusion matrix of a realization. Each section shows the number of cells classified in each category. Dark blue sections indicate the number of cells correctly categorized. **(B)** Venn diagram of the relevant genes found on the five realizations in the resampling process, numbers stand for genes shared across realizations. Eight genes were found in cross-validation analysis. **(C)** SHAP plot of one realization. In the figure, we depicted the first twenty genes with higher contribution to the classification of moderate and severe patients. We have ordered the genes from high to low relevance from top to bottom. Blue and red colors represent low and higher gene expression, respectively. Larger positive values in the SHAP axis set the gene relevance to classify severe patients, whereas negative values set the gene relevance for moderate patients. **(D)** Validation confusion matrix. Each section shows the number of cells classified in each category. Dark blue sections indicate the number of cells correctly categorized.

Severity classification is crucial to apply a treatment tailored for every patient and reduce overall mortality. This challenge summons the need for a severeness biomarker to classify COVID-19 patients efficiently and improve their prognosis. Although we have shown that the samples have a heterogeneous composition in their cell-type population, in this section we survey possible genetic biomarkers of the disease severity. To this aim, we applied a machine-learning algorithm to construct a classifier of the clinical output from scRNASeq and identify a set of genes with relevance to classify the single-cell RNAseq samples in severe and moderate data. We followed four main steps: selecting the data for training and testing the model, building the classification model (Extreme Gradient Boosting, XGBoost), assessing the model performance, and identifying variables that contribute to the classification. As a result, our machine-learning model correctly classified cells from moderate and severe patients with an accuracy of 0.98 for testing data (**Figure 2A**). To verify the reproducibility of this finding, we assessed its robustness by constructing an ensemble of machine-learning models with different sets of training and testing datasets. Specifically, we used a k-fold cross-validation method, consisting of a resampling procedure for dividing the entire data into distinct groups of train and test data sets. First, we split the whole data into five random equivalent proportions. Afterward, we iteratively resample the data by selecting one proportion as the testing data and the rest as training data. We repeated the resampling process five times, each one selecting a different set of data for training and testing. We built an XGBoost model in each realization and evaluated its performance through the area under the ROC curve (AUC). The average AUC over the ensemble was 0.97, showing that the model was robust in selecting the training and testing datasets (**Figures S3, 4**). Besides, by comparing the results obtained from the cross-validation method, we identified a signature of eight genes that always appeared in the set of relevant genes to classify moderate and severe patients, independently of the data resampling (**Figure 2B**). These genes were RPS26, MT-ATP8, CCL2, MT-ND4, MALAT1, APOC1, CXCL8, and NUPR1. To explore the relevance of these genes in the classification process, we selected one of the XGBoost models (see *Methods*) and calculated the SHAP value for each gene, a numeric value that ranges from positive to negative values and quantify the level of contribution of the gene into the classification (19). In our case, a gene contributes to classify moderate patients when its SHAP value is negative. In contrast, the gene contributes to classify severe patients when its SHAP value is positive. It is important to note that one gene can have different SHAP values accordingly its gene expression changes from low to high (**Figure 2C**). For instance, MT-ND4 contributes to classify severe patients when it has high expression levels (positive region in the SHAP values) (**Figure 2C**). However, MT-ND4 contributes to classify moderate patients when it has low expression (negative SHAP values). Overall, there is an insight into each gene over and subexpression tendency associated with classifying moderate and severe patients. The set of genes with a high average expression level in severe patients were RPS26, CCL2, MT-ND4, and CXCL8. Otherwise, MT-ATP8, MALAT1, APOC1, and NUPR1 belong to the set of genes with a low average expression level in severe patients (**Figure 2C**). Thus, we postulate that this set of genes and their expression levels can classify patients with severe and moderate severity from their single-cell gene expression profiles.

To verify the performance of the XGBoost model in classifying cells in another dataset, we evaluated the performance of the XGBoost model over an entirely new and recently published dataset of scRNAseq for BALF samples of 9 patients with moderate and severe responses (21). This dataset represents a total of 41573 cells obtained from 6 severe and 3 moderate patients. The model classified these cells with high accuracy from their gene expression profile (**Figure 2D**). Besides, among the genes that are part of the gene signature, there are included quality-associated genes (MTR, RPS, and RPL), non-coding genes (MALAT1), and interferon-stimulated genes (ISG). Reports indicate that the expression of ISG has an inherent dynamic (30). In order to evaluate the robustness of our computational pipeline, we reconstructed and assessed the performance of a new classifier that excluded MALAT1, ISG, MTR, RPS, and RPL. Two criteria mainly selected this set of genes, first, by literature research. Second, genes obtained from the interferome database including type I, II, and III interferons (IFN) regulated genes manually curated from publicly available microarray datasets (31). Having defined the set of genes to be excluded in the analysis (32 in total, **Table S3**), we trained a new machine-learning model and evaluated its performance as previously described. We concluded that our reduced model presents high accuracy to classify severe and moderate cells from BALF samples (**Figure S5**). Notably, when assessing its performance with the new set of scRNAseq BALF samples, the model classified severe and moderate cells in an accurate way (**Figure S5**).

In summary, our machine-learning approach allowed us to construct a computational model helpful for classifying severe or moderate responses in BALF samples from COVID-19 patients, which in the clinical setting would help make a series of decisions to act more efficiently and quickly. In this section, we have built a machine-learning model that differentiates severe from moderate patients from a bulk population without considering the detailed description of the population heterogeneity in the samples. In the following sections, we explore how COVID-19 infection affects transcriptional profiles of different subpopulations in different patients. Using these results, we examined the association between the expression profile in cells with the disease severity among patients and described their landscape of molecular mechanisms underlying COVID-19 infection.

### 3.3 Epithelial Cells

**Figure 3:**
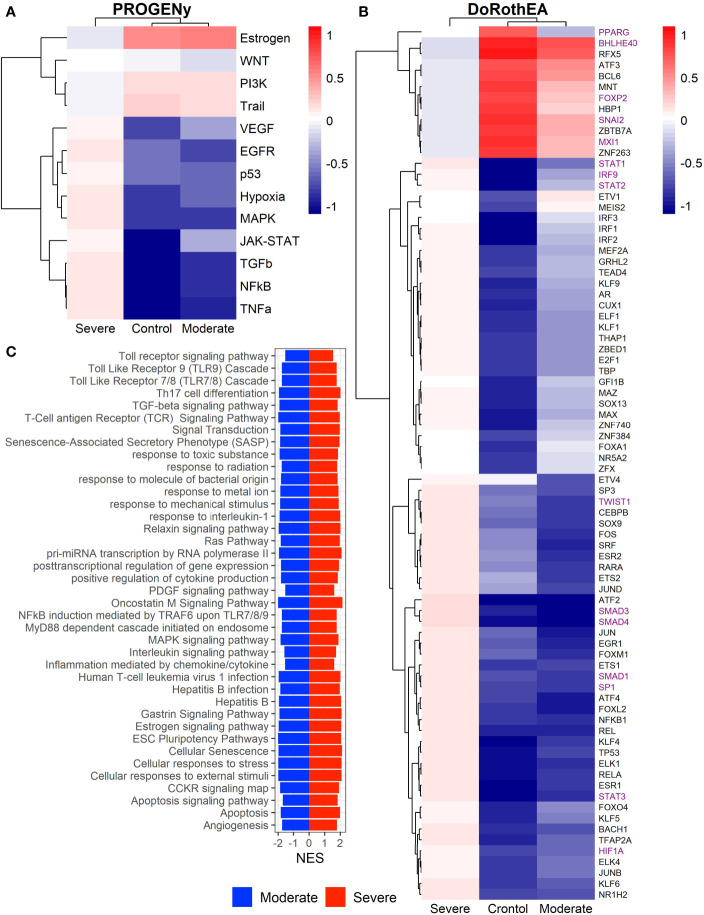
Pathways and transcription factors dysregulated on epithelial cells among patients. **(A)** Changes in the pathways activation/inactivation identified by PROGENY analysis. White boxes indicate no change of the pathways in the group of patients respectively. **(B)** Transcription factors inferred by DoRothEA algorithm. Colorbar is related to activation/inactivation values. **(C)** Pathway Enrichment Analysis, the normalized enrichment score (NES) value represents the activity status within the disease severity conditions. Blue and red bars relate NES values for the moderate and severe patients, respectively.

Epithelial cells have a crucial role during COVID-19 infection, contributing to the disease severity through a dysfunctional response to viral infection (3, 4, 28, 33–35). SARS-CoV-2 induces transcriptional signatures in human lung epithelial cells that promote different symptoms (29). However, the pathways and transcriptional factors regulating these expression patterns are unknown, limiting the understanding of the effects of the infection on these cells. Therefore, we used single-cell RNAseq data to infer pathway activity using an algorithm called PROGENy (24). We found differences among patients within disease severities in essential pathways for many cellular processes (**Figure 3A**). For instance, we discovered that JAK-STAT, NFKβ, TGFβ, TNFα, and Hypoxia pathways are central players underlying and differentiating the COVID-19 pathophysiology, as their tendency is opposite between moderate and severe patients. Their dysregulation affects the alveolar cells that shape the innate and adaptive immune system (36). As can be seen, estrogen signaling is overactivated for moderate patients. Since samples on all groups were from female and male patients, we assumed the estrogen signaling activation was not gender derived. Moreover, this pathway has crosstalk with others that might cause the activation.

To better comprehend the regulatory mechanisms underlying previous pathways and their differences among patients, we inferred the activity of the potential transcriptional factors considering the expression of their targets. DoRothEa algorithm helps with this aim using gene expression data and a predefined gene set containing TF-target interactions (24). As a result, we obtained insights into opposite processes within patients. For instance, PPARG shows low activity for COVID-19 patients (**Figure 3B**). Although, PPARG in airway epithelia is necessary for typical structure and function (37). In addition, it plays a role in the monocyte/macrophage-mediated inflammatory storm (38); PPARG activation might reflect the dysregulation caused by the SARs-CoV-2 despite the severity.

Moderate patients have BHLHE40 overactivated and SP1 and TWIST1 inactivated; severe patients showed an inverse activation/inactivation pattern. TWIST is negatively regulated by BHLHE40 and positively by SP1, and the three of them are involved in the epithelial-to-mesenchymal transition (EMT) (39). Besides, MXI1 has the same activation fashion as BHLHE40, and it is an antagonist of Myc. Myc and EMT have been directly associated (40). TWIST1 and HIF1A are slightly overactivated in severe patients; both are necessary to develop EMT and Endothelial Mesenchymal Transition (EndMT) (41). In addition, severe patients have inactivated FOXP2, whose inhibition induces EMT and activates TGFβ signaling (42). EMT and EndMT might be central to COVID-19 pulmonary fibrosis, mainly developed on epithelial cells. As a part of the EMT and EndMT, the basement membrane underlying endothelial cells gets disrupted, facilitating the migration of cells, causing pulmonary fibrosis, endothelial damage, and pulmonary edema, worsening the severity of the disease (41). These effects are observed in lung tissues from post mortem patients who died from COVID-19 (43, 44). Furthermore, we hypothesize these processes could be related to the immune evasion processes initiated by the infection. Consequently, severe patients might trigger EMT and immune evasion contrary to moderate patients. Contrastingly, moderate patients overactivated SNAI2, a crucial TF to the EMT development (45). Thus, the EMT contributes to COVID-19 pathophysiology (46), a condition that does not exclude moderate patients. Moreover, the severe patients have activation of the TGFβ pathway suggesting an instauration of the ETM (**Figure 3A**). TGFβ pathway is triggered once the EMT is set (46).

As for the STAT1, STAT2, and STAT3 showed lower activity in moderate than severe COVID-9 patients (**Figure 3B**). STATs belong to the JAK-STAT signaling pathway that also led to a lower activity on COVID-19 patients. IFN-induced STAT1/STAT2 nuclear translocation is the essential step for antiviral signal transduction. During COVID-19, SARS-CoV-2 nucleocapsid (N) protein binds to STAT1/STAT2 with the downstream kinases and inhibits their phosphorylation (47). Besides, this virus also promotes STAT1 proteolysis and retains the import factors of STAT1 at the ER/Golgi membrane, blocking the expression of STAT1-activated genes that establish an antiviral state (33, 48, 49). Which seems counterintuitive because moderate patients inactivate STAT1/2/3. Also, SARS-CoV-2 disrupts IFN induction by preventing the transport of IRF3 and STAT1 into the nucleus. Moreover, deficiency of STAT1 demonstrated a markedly worsening pulmonary disease with inflammation of small airways and alveoli (50). Several convergent findings suggest STAT1 as a putative cause of the cytokine storm observed in the most severe cases of COVID-19 (34). Although inhibition of STATs is necessary to enhance the disease pathophysiology disrupting IFN signaling, it is contrary to our findings. Moreover, to clarify the differences within severe and moderate patients, we employed a gene-set enrichment analysis (GSEA) over the TFs list from Dorothea to systematically explore the potential phenotype of the epithelial cells (**Figure 3C**). Under the enrichment analysis, moderate and severe patients have opposite phenotypes in the same pathways, inactivation/activation for the moderate/severe patients according to their normalized enrichment score (NES). Severe patients have activation of the TLR pathway; activation of TLR leads to the secretion of pro-inflammatory cytokines such as IL-1, IL-6, TNFα, as well as IFNI (51). SARS-CoV-2 spike protein in epithelial cells promotes IL-6 trans-signaling by activating the AT1 axis to initiate coordination of a hyper-inflammatory response (52). Consistently, interleukin pathways were positively enriched for severe patients and negatively for the moderate ones. Additionally, severe patients showed a positive enrichment of TGFβ, MAPK, and angiogenesis pathways. In severe COVID-19 patients, TGFβ is associated with an uncontrolled immune reaction, in which STAT3 and SMADs genes take part (53). Moderate patients have inactivation of several SMADs (SMAD1, SMAD3, and SMAD4) and STAT3 (**Figure 3B**). Under the multiple functions of the TGFβ pathway, it is associated with the ETM *via* ERK/MAPK pathway (54). Therefore, severe patients showed a tendency to be affected by cytokine hyperactivation and a possible EMT state.

**Figure 4:**
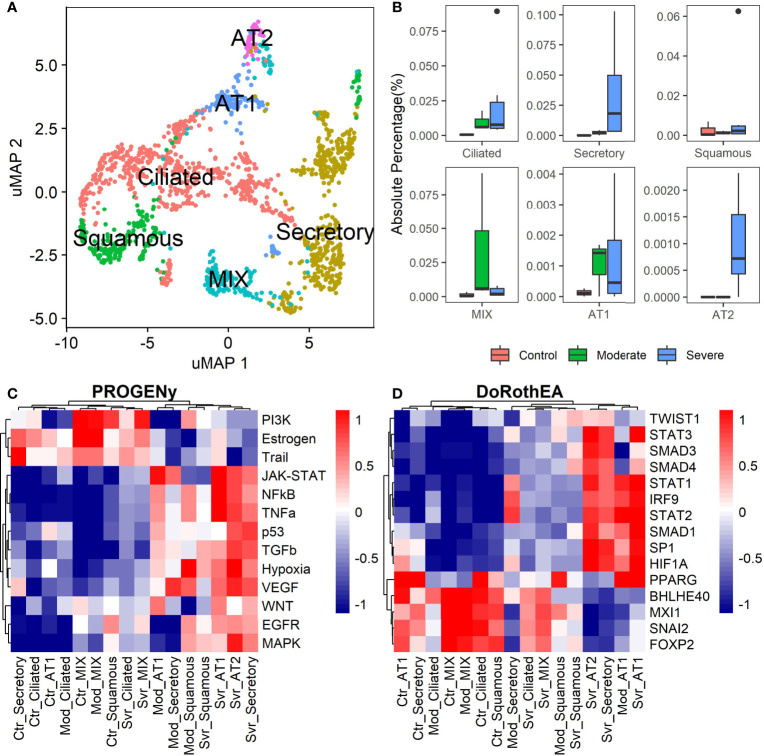
Epithelial cells diversity analysis. **(A)** Umap projection of epithelial cells data showing the five cell subtypes identified based on biomarkers: ciliated, secretory, squamous, alveolar type I (AT1), and alveolar type II (AT2) (**Table S2**). **(B)** Proportions of the epithelial subtypes among healthy controls, moderate and severe patients. **(C)** Pathways activation/inactivation analysis with PROGENY for the epithelial cells subtypes considering health status. **(D)** Activation/inactivation analysis considering health status for the TFs discussed on the whole-epithelial analysis (TFs denoted in purple along with **Figure 3B**). Colorbar is related to activation (red) and inactivation (blue) values. Ctr, Mod, and Svr stand for healthy control, moderate and severe patients.

The previous analysis considered epithelial cells as a whole, describing the general behavior according to the disease severity. Moreover, the lungs have diversity in their epithelium composition related to the tracheobronchial tree. To evaluate the role of the different epithelial cells in the COVID-19 progression, we reclustered the data of these cells. We identified five epithelial subtypes projected in 12 clusters in the uMAP space (**Figures 4A** and **S6A**). We typified the epithelial subtypes using 14 marker genes (**Table S2**) comprising ciliated, secretory, squamous, alveolar type I (AT1), and alveolar type II (AT2) cells. Additionally, we identified one cluster that expressed AT2 and secretory markers. Experimental evidence showed that secretory cells expressed SFTPB and SFTPC (AT2 markers) (55, 56). Moreover, we named this group MIX due to the possible implication in their functionality. Overall, severe patients have more percentage of secretory and AT2 cells (**Figure 4B**). Reports indicate that AT2 cells exhibit a high proliferation rate at early-phase pneumonia marked by the expression of MKI67 (57). Pneumonia is a complication of COVID-19. Therefore, to understand the higher proportion of epithelial cells from severe patients, we evaluated the proliferation marker MKI67 expression (**Figure S6B**). We cannot know the pneumonia phase among severe patients. Only some severe epithelial cells expressed MKI67 suggesting a possible prior proliferation stage that explains the epithelial proportions among controls and patients. Moreover, as the epithelial cells are the primary entrance of SARS-COV-2 to the human body, we observe an alteration in their balance related to the disease severity, possibly affecting their role. To evaluate the triggered processes, we inferred the pathway activity using PROGENy. Comparing the activity of the pathways between the whole-epithelial analysis (**Figure 3A**) and the separated-epithelial analysis (**Figure S6C**), we observed that the tendency in moderate patients is related to the ciliated and mix cells, as for the severe patients, their pathways activity is correlated to AT1, AT2, and secretory cells. Then, we evaluated if the activation of the pathways is related to the severity of the patients (**Figure 4C**). We found AT1, AT2, and secretory cells from severe patients overactivated JAK-STAT, NFKβ, TGFβ, TNFα, and Hypoxia pathways. Congruently with these results, dysregulation of these pathways affects the alveolar cells that shape the innate and adaptive immune system (36) that might be worse for severe patients. Subsequently, taking the TFs highlighted in the whole-epithelial analysis (**Figure 3B**), we evaluated their activity for the epithelial cells subtypes (**Figure S6D**). Interestingly, we observed the activation of TFs associated with EMT all over the lung cells, suggesting that different lung compartments support the EMT and not only the alveolar. To illustrate, PPARG is activated only in AT1 cells, promoting a pro-inflammatory state leading to alveolar fibrosis. As for AT1, AT2, and secretory cells, overactivated SMADs, HIF1A, and STATs genes. We evaluated the same TFs for the different epithelial cells split by healthy controls and patients (**Figure 4D**). Complementary, these results denote that AT1 cells from moderate and severe patients along with AT2, and secretory cells from severe patients overactivated SP1, SMADs, STATs, TWIST, HIF1A, and IRF genes. As already discussed, these genes are fundamental to EMT initiation and progression. Moderate patients seem to have the condition for the EMT as well, but with only AT1 cells. The TFs that inhibit the EMT are activated mainly in all moderate cells. Ciliated and MIX cells overactivated SNAI2 for severe patients, suggesting that several lung structures help EMT. Considering all the above, we hypothesize that cytokine activation and ETM could be related to the immune evasion processes in the SARS-CoV-2 infection. In conclusion, our study suggests that it is necessary to associate EMT and EndMT with the cytokine storm observed in this infectious disease to further understand the mechanisms in the context of COVID-19 to prevent and treat pulmonary fibrosis appropriately.

### 3.4 Monocyte Heterogeneity in COVID-19 Patients

**Figure 5:**
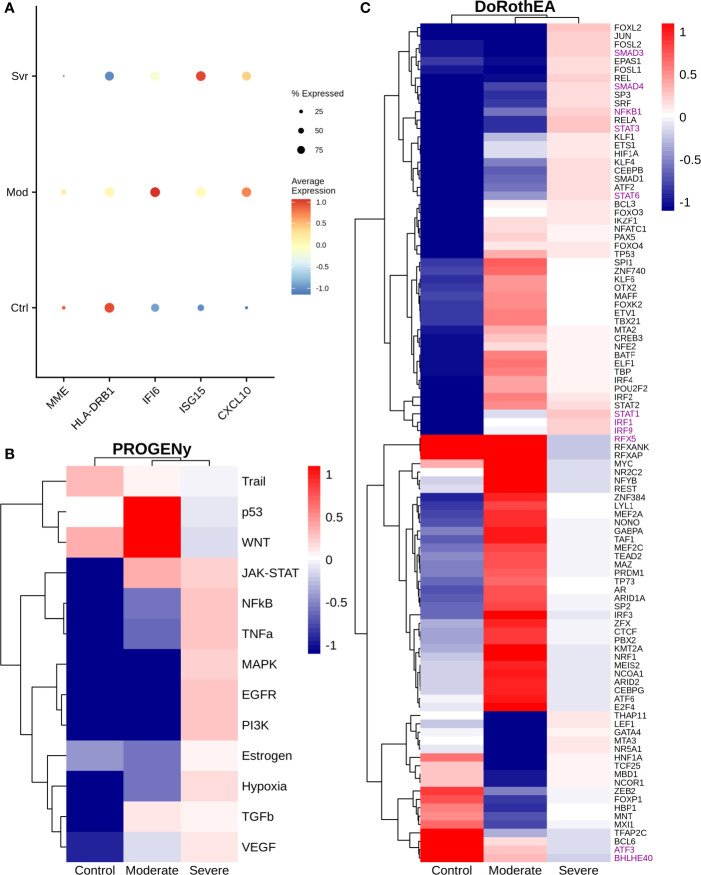
Gene expression and inferred transcriptional factor activities of monocytes among patients. **(A)** Expression of several marker genes across patients, the point size and colorbar are related to the percentage of cells that express a gene and the average expression, respectively. **(B)** Predicted pathway activity among patients. **(C)** Transcriptional factor activity of monocytes inferred using single cell data. Colorbar is related to activation (red) and inactivation (blue) values.

Monocytes are cells of the innate immune system circulating in the blood that extravasate to the tissue once an inflammatory process in the lungs is activated. They engage in inflammatory processes, antigen presentation, among other functions (58, 59). COVID-19 severity is associated with an intense inflammatory response of the cells caused by the cytokine storm, plus an inadequate immune response (60, 61). Therefore, we evaluated some genes associated with deficient immune response and inflammation on monocytes across different disease severities (**Figure 5A**). For instance, we observed a higher expression of HLA-DRB1 in moderate than severe patients. The HLA-DRB1 gene is part of a family of genes called the human leukocyte antigen (HLA) complex. The HLA complex helps to identify proteins from viruses and bacteria. Together with HLA-DRA, HLA-DRB1 forms a functional protein complex called the HLA-DR antigen-binding heterodimer. This complex displays foreign peptides to the immune system to trigger the immune response. Severe cases of COVID-19 have low expression of HLA-DR in blood monocytes (62–64). In concordance, our results suggest that the immune system of moderate patients led to an efficient response to COVID-19 infection. Therefore, the lack of expression of these HLA genes in severe patients may indicate an inadequate immune system response. Moreover, we also observed changes in IFI6 and ISG15 genes. IFI6 has a higher expression in moderate respect to severe COVID-19 patients, whereas ISG15 behaves viceversa. These genes are IFN-stimulated genes correlated with an inflammatory response. On blood monocytes, low HLA-DR and high IFN-stimulated genes have been associated with severe cases of COVID-19 at later stages, indicating a prolonged activation of monocytes in severe COVID-19 (64). In concordance, our results showed a similar expression pattern on lung monocytes. Additionally, MME exhibited a higher expression in moderate than severe patients (**Figure 5A**). This gene is involved in the differentiation of monocytes to the inflammatory associated M1 macrophage. We hypothesize that some of the monocytes in moderate patients differentiated to M1 macrophages, liberating pro-inflammatory cytokines and other peptides to eliminate the virus (65). Moreover, MME cut many active peptides useful in the inflammatory process, including angiotensin II (66). Angiotensin II is elevated in patients with severe COVID-19, strongly associated with viral load and lung injury (67). Consequently, the high expression of MME on moderate patients could lower angiotensin II levels and inhibit severe complications of COVID-19, thereby protecting the patients. In conclusion, results showed a possible dysregulation into the monocytes during the infection correlated with the severity of the disease, indicating the response of these cells is essential to determine the disease severity.

Specific pathways highly regulate the immune response. Hence we evaluated pathway dysregulations correlated to disease severity. To this end, we used PROGENy, a method that infers pathway activity from gene expression data, in combination with our single-cell RNAseq dataset (24). As a result, NFKβ, TNFα, and MAPK exhibited lower activity in moderate than severe patients (**Figure 5B**). Notably, these pathways are essential for immune response. This finding suggests that monocytes are only being recruited at this stage but do not have a pro-inflammatory function. On the other hand, TRAIL showed almost a similar activity in moderate and severe patients. In monocytes, the TRAIL pathway is involved in activating pro-apoptotic regulators (68), suggesting that monocytes are not dying in the lungs of COVID-19 patients. Additionally, to understand the mechanisms behind monocyte dysregulation, we explored specific transcription factors (TF) activity using the algorithm called DoRothEa. Under the comparison between moderate and severe patients, several TFs involved in controlling pro-inflammatory genes showed a decrease in moderate patients, such as STAT1/3/6, SMAD3/4, and NFKB1 (**Figure 5C**). In addition, we observed a lower activity of IRF1 and IRF9 in moderate patients. IRF genes regulate interferon genes once the pattern recognition receptors detect viral RNA (69). On the one hand, IRF1 is involved in the polarization of monocytes to an M1 macrophage by enhancing the expression of inflammatory cytokines, developing a dysregulation of macrophage behavior, and developing hyper-inflammation (70). On the other hand, IRF9 regulates interferon gene expression and activates a type I interferon response (71). Hence its low activity causes a delayed response for most COVID-19 patients. Complementary, RFX5, ATF3, and BHLHE40 showed a higher activity in moderate than severe patients (**Figure 5C**). These results suggest that among patients, moderate patients have a higher differentiation of monocytes-macrophages, inhibition of the secretion of IL-10 (BHLHE40), activity of MHC class II genes (RFX5) and regulation of the immune response by controlling the expression of metalloproteinase (ATF3) that in severe patients (72–74).

### 3.5 Macrophage Heterogeneity Among COVID-19 Patients

**Figure 6:**
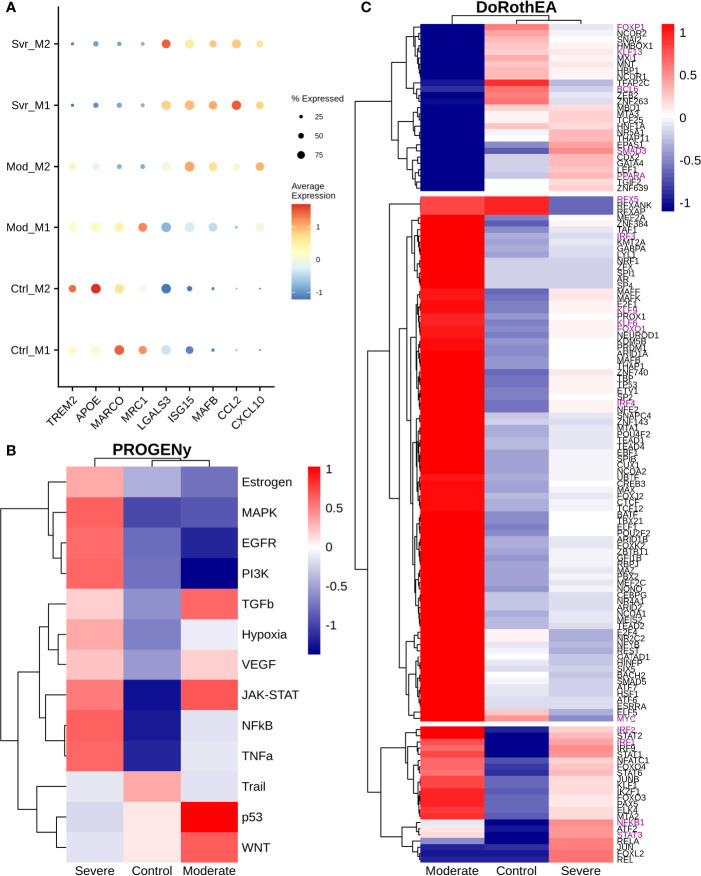
Differential macrophage activity among patients. **(A)** Expression levels of inflammatory and fibrotic related genes, point size and colorbar represent the percentage of cells that express a gene and the average expression, respectively. **(B)** Pathway differential analysis among patients. **(C)** Transcription factors altered on macrophages among disease severity. Colorbar is related to activation/inactivation values. M1 stands for macrophage type I and M2 for macrophages type II.

Macrophages are abundant in the lungs during COVID-19 infection (28). Based on their cytokine secretion, macrophages exert an anti-inflammatory or proinflammatory activity. Therefore, we explored the expression levels of genes typically associated with a hyper-inflammatory microenvironment, impairment of interleukin secretion, and enhanced anti-inflammatory functions associated with COVID-19 symptoms. Macrophage subpopulations were split based on (1). We found TREM2, APOE, MARCO, and MRC1 that showed higher expression in moderate M1 than moderate M2 macrophages. Interestingly, severe patients exhibited an absence or low expression of these genes (**Figure 6A**). Otherwise, LGALS3 showed high expression on severe patients and low expression on moderate patients and healthy controls. The expression of TREM2 avoids macrophage apoptosis and enhances the secretions of pro-inflammatory cytokines (75). TREM2-APOE-LGALS3 is usually correlated with pro-fibrosis and heightened inflammatory response, inducing IL-6 secretion (76, 77). Additionally, ISG15 and MAFB exhibited a low expression in moderate M1 macrophages and high expression in moderate M2 macrophages (**Figure 6A**). MAFB promotes an anti-inflammatory function in inflamed lungs leading to M2 macrophage polarization (78, 79). Interestingly, the expression of MAFB in macrophages is associated with an impaired type I interferon response in chronic hepatitis virus (80). Finally, CCL2 and CXCL10 genes lacked expression in moderate patients; only CXCL10 had higher expression in moderate M2 macrophages. However, severe COVID-19 patients highly expressed both genes compared to healthy controls (**Figure 6A**). In addition, CCL2 and CXCL10 showed a slightly higher expression in severe patients than in moderate patients. CXCL10 and CCL2 are important chemoattractants for monocytes and macrophage recruitment to the inflamed tissues. Nevertheless, these expression patterns suggest a difference between macrophages of moderate and severe patients. The reason underlying these differences remains unclear as many genes related to inflammation and fibrosis have controversial behavior among patients. Hence, to suggest the potential role of macrophages on disease pathogenesis, we evaluated the altered pathways.

To infer pathway activity of macrophages using single-cell data, we used PROGENy as the previous sections (24). Accordingly, we observed the inactivation of NFκB, TNFα, and MAPK in moderate compared with severe patients, as in monocytes of previous sections (**Figure 6B**). Additionally, we noted some differences with monocytes data. For instance, we observed activation of EGFR, TNFα, and MAPK in severe patients. The differences in the pathway activity between moderate and severe patients could be responsible for the diverse symptomatology among patients. Activation of EGFR correlates with the expression of anti-inflammatory cytokines, enhancing the expression of TGFβ expression (81). Additionally, TNFα and MAPK promote the secretion of IL-6, contributing to the cytokine storm in COVID-19 patients (82, 83). Therefore, to explore the possible genetic regulatory mechanisms underlying these altered pathways, we infer the transcription factor activity using DoRotheA. As the initial step, we reclustered the macrophages cells based on their activity of TFs. Accordingly, cells from patients (moderate and severe) clustered together, apart from cells of healthy controls (**Figure S7**). Briefly, we observed three sets of transcription factors with inverse activation (**Figure 6C**). On the one hand, the first set of transcription factors exhibited low activity in moderate patients and mixed behavior in severe patients and healthy controls. Forkhead transcription factor (FOXP1) has low activity in moderate patients and no activity in severe patients. The downregulation of FOXP1 in macrophages is associated with the secretion of IL-12 and TNFα, cytokines implicated in eliminating pathogens (84). Kruppel-like factor 13 (KLF13) has low activity in moderate patients and high activity in severe patients. Low expression of KLF13 is implicated in diminishing pro-inflammatory and enhancing phagocytic activity in macrophages, a necessary balance in maintaining an efficient immune response (85, 86). BCL6 has low activity in severe and moderate COVID-19 patients; its loss implies hyper-proliferation, followed by the expression of STAT3 to secrete IL-6, contributing to the cytokine storm in severe COVID-19 patients (87). SMAD3 has low activity and is associated with diminishing the secretion of TGFβ, implicated in decreasing the secretion of cytokines promoting the cytokine storm in severe patients (88). As well, SMAD3 activates STAT6, which induces a fibrotic response in macrophages (89). PPARA has high activity in severe patients and low in moderate patients. It is associated with mitigating an inflammatory response (90). The second set of transcription factors has high activity in moderate patients and low activity in severe COVID-19 patients. RFX5 is associated with the MHC II response, macrophage proliferation, and effective immune response by presenting antigen to CD4+ T cells (91, 92). IRF3 activation is implied with a dual role. It can induce a type I interferon response (93), and it secretes pro-inflammatory cytokines contributing to the cytokine storm (94). Meanwhile, expression of IRF4 is associated with the secretion of IL-4 and IL-10 which are anti-inflammatory cytokines that may be important to reduce the damage of the cytokine storm in moderate patients and not develop in severe COVID-19 symptoms (95). Similarly, Kruppel-like factors 4, 6, and 9 (KLF4, KLF6, and KLF9) exhibited high activity on moderate patients, in contrast to their relatively low activity on severe patients. These KLFs mount inflammatory and fibrotic responses in low oxygen microenvironment enhancing cytokine secretion, contributing to cytokine storms typical of severe patients (96, 97). Likewise, the hypoxia pathway (**Figure 6B**) showed an inferred high activity on severe patients, explained by the respiratory distress, low oxygen saturation, and pulmonary lesions caused by the disease. FOXO1 activates IRF4 (98). Lastly, from the second set, MYC is associated with alternative macrophage activation, secreting anti-inflammatory cytokine in moderate patients, which may help balance the damage held by the pro-inflammatory cytokines; this function is not present in COVID-19 patients (99). In the last set, moderate and severe patients Overactivated the TFs, except NFKB1 and STAT3, whose expression is divergent between them. Many of these TFs correlate with the secretion of interleukins involved in the induction of inflammation. STAT1 and STAT2 have higher activity in moderate patients than severe patients, suggesting a possible delay in the interferon response in severe COVID-19 patients. Meanwhile, STAT6, an anti-inflammatory TF, has higher activity in moderate patients and might diminish the action caused by the pro-inflammatory cytokines in the lungs. On the other hand, IRF1 and IRF2 are regulators of the interferon response belonging to the third category. This result suggests that some macrophages may regulate the interferon response, which can help eliminate the virus from the lungs. Finally, the moderate patient group did not activate NFKB1 and low activity of STAT3. In severe cases both transcription factors are activated. NFκB activation is mainly involved in the secretion of pro-inflammatory cytokines (100), whereas STAT3 releases anti-inflammatory cytokines, proposing a high abundance of pro-inflammatory cytokines. In moderate patients the action of anti-inflammatory cytokines are balancing the action of pro-inflammatory cytokines, meanwhile in severe patients the balance is towards the action of pro-inflammatory cytokines causing the cytokine storm in COVID-19 patients. Overall, these results indicate macrophages of severe patients have low effectiveness against the infection followed by an enhanced inflammatory response, causing severe and critical symptoms. In contrast, moderate patients showed macrophages with suspected high efficiency and controlled inflammation input, diminishing the disease severity. In conclusion, we suggest that an appropriate macrophage response could limit the disease severity.

### 3.6 NK Cells

**Figure 7:**
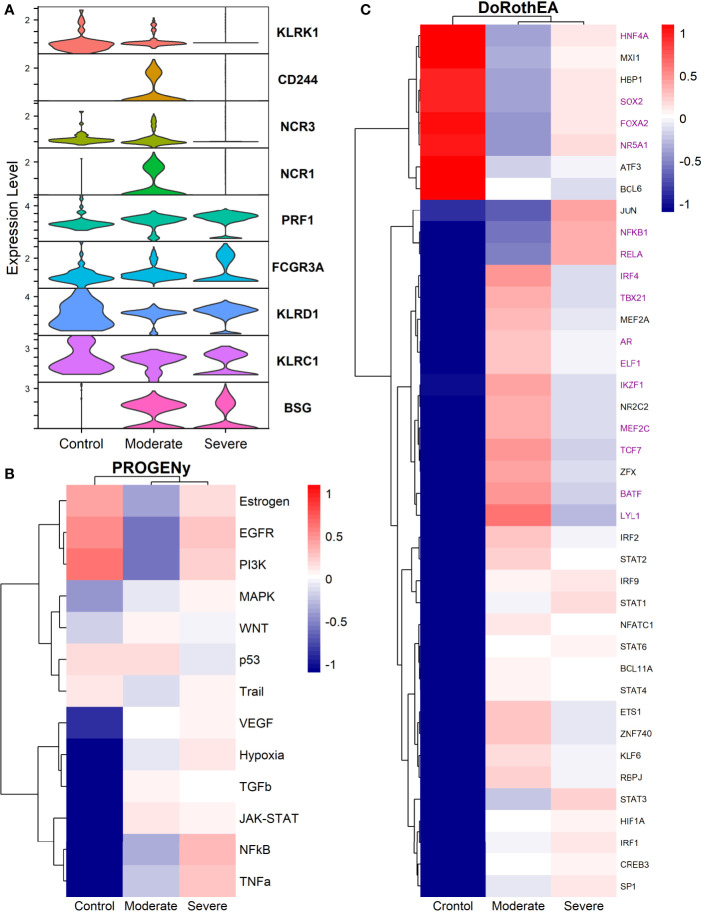
TFs associated with the disease severity in NK cells. **(A)** Violin plots for several markers for the healthy controls, moderate and severe patients. **(B)** Resulting Heatmap from PROGENy analysis. Colorbar sets the activation (red) and inactivation (blue) values. **(C)** Resulting Heatmap from DoROthEA analysis.

Natural Killers (NK) cells take part in the defensive frontline against viruses. They induce self-destruction of virus-infected cells by apoptosis. Additionally, NK cells set the maturation of dendritic and T cells. Studies have reported that positive SARS-CoV-2 patients have decreased circulating NK cells (101). Moreover, given our results, this is only true for severe patients in the alveolar compartment. Moderate patients have a higher NK count compared to severe patients and healthy controls (**Figure 1C**). The immune role of NK cells has a balance between activating and inhibiting germline-encoded receptors; this dual functionality ensures protection against pathogens and prevents auto-immune responses. To evaluate the activation state of NK cells among healthy controls and patients, we plotted the expression of activating receptors related to infection and cellular distress (**Figure 7A**). KLRK1 (known as NKG2D), CD244, NCR3 (known as NKp30), and NCR1 (known as NKp46) were preferentially expressed in moderate patients and healthy controls. Through the co-activation of these receptors, the NK cells mount an effective response to induce pathogens killing (102, 103). The expression of PRF1 (perforin) and FCGR3A (CD16) might indicate that NK cells from moderate patients undergo their activation by two mechanisms: 1) direct lysis of target cells through cytotoxic degranulation by perforin, and 2) the detection of antibody-coated target cells (102, 104). In COVID-19, the expression of the inhibitory marker KLRC1 (also known as NKG2A) leads to decreased NK cells cytotoxic activity by affecting the IFNγ and TNFα pathways (105). KLRC1 is expressed preferentially in severe patients, which suggests a possible non-responsive state. However, moderate patients might undergo inactivation. Finally, both groups of patients expressed BSG, there is evidence that BSG mediates SARS-CoV-2 entering host cells by endocytosis (106). Previous markers pose the role of NK cells, but more analysis is needed to associate a function.

Functional pathways analysis with PROGENy showed the activation of pathways among patients and controls (**Figure 7B**). Furthermore, Severe patients shared an overactivation with healthy controls of the Estrogen, EGFR, and PI3K. The estrogen pathway has crosstalk with the NFkB signaling; NFkB and TNFα had been related to the dreadful cytokine storm in COVID-19 (107), and they are overactivated only for severe patients. TNFα activates the NFkB pathway *via* the non-canonical pathway associated with long-lasting pro-inflammatory mediators production (107). In addition, the SARS-CoV-2 triggers EGFR and PI3K pathways. Under these activations, a profibrotic response is released (108). BSG (also known as CD147) activates the PI3K pathway, a SARS-CoV-2 receptor mediating the virus entry and overexpressed patients (**Figure 7A**). As well, once the virus binds to the ACE2, it is internalized by endocytosis through a clathrin-mediated pathway regulated by the PI3K/AKT signaling (109). Therefore, NK cells from severe patients seem to be coadjuvant in the cytokine storm progression, virus internalization, and respiratory failure. Contrastingly, moderate patients have inactivation of previously described pathways (**Figure 7B**), setting a non-harmful response but still active according to their markers (**Figure 7A**).

By the use of Dorothea, we explored regulatory mechanisms underlying these pathways and differences among patients (**Figure 7C**). Severe patients showed alterations in the cell cycle, taking the TFs inactivated for moderate patients and overactive for severe patients and healthy controls. They increased cytotoxicity, particularly within the interaction between NK and T cells. For instance, the activation of HNF4A and SOX2 is related to abnormal cell proliferation and mucus hyper-secretion (110, 111). MXI1 overactivation has an antagonist role to MYC (112, 113). FOXA2 regulates Treg cell suppressive function and inflammation (114, 115). Finally, NR5A1 may be related to the cytotoxicity, proliferation, and cytokine production of E2-mediated NK cells (116). Although previous genes are described in their effect on T cells, NK cells influence the T cells functionality at different stages. At the early stages of T cell activation, NK cells shape the ensuing size and quality of the T cell responses. Additionally, they influence T cells clonal expansion, immune memory formation, and the interaction with Treg (117).

TFs only activated for severe patients include JUN, NFKβ1, and RELA; they interact *via* NFKβ signaling. RELA and NFKβ1 form the complex NFKβ directly related to an inflammatory response of innate cells, and specifically for NK, the complex regulates the proliferation (118, 119). Otherwise, AP-1 is a dimeric complex that consists of members of the JUN and Fos families. AP-1 regulates gene expression in viral infections, and it is a target of SARS-CoV-2 affecting their response (121). JUNB is expressed mainly in patients cells (**Figure 7A**). Therefore, NK cells of severe patients seem to be altered in the NFKβ pathway being overactivated by several stimuli.

Based on the activated transcription factors on moderate NK cells, we suggest two primary immune responses. First, cells from moderate patients elicited a controlled immune response to promote viral clearance without a possible cytokines hyper-activation. IRF4, TBX21, and BATF have been identified as crucial players to mediate NK cytotoxicity and interactions with other immune cells within inflammatory tissues (122). However, IRF4 takes part in an impaired response signature in HIV infection (123, 124). BATF regulates lymphoid homeostasis, and their subexpression leads to inflammation and inadequate innate response (125). LYL1 also is essential for T cell homeostasis, development, and maintenance (123). ELF1 inhibits virus replication, and their antiviral response is distinct from the interferon signature providing another innate host response, independent from the action of type I interferons (126, 127). NK cells lacking IKZF1 are hyper-reacting and have impaired viral contention (128). MEF2C deficiency was associated with profound defects in the production of B cells, T cells, and NK cells (129). Finally, TCF7 prevents NK self-destruction by regulating granzymes (129, 130). The second observed response according to the activated TFs is unclear and might be unfavorable for moderate patients. AR suppresses IL-12A expression lowering the efficacy of NK cell cytotoxicity (126). NR2C2 is an integrant of an axis identified to promote abnormal T cell activity on lupus (131). Our results suggest a complex responsive state in NK cells predominantly orientated to an antiviral response. Nevertheless, previously mentioned mechanisms might be off in severe patients, indicating possible non-responsive NK cells to the virus.

### 3.7 Dendritic Cells Function Regulation According to Disease Severity

**Figure 8:**
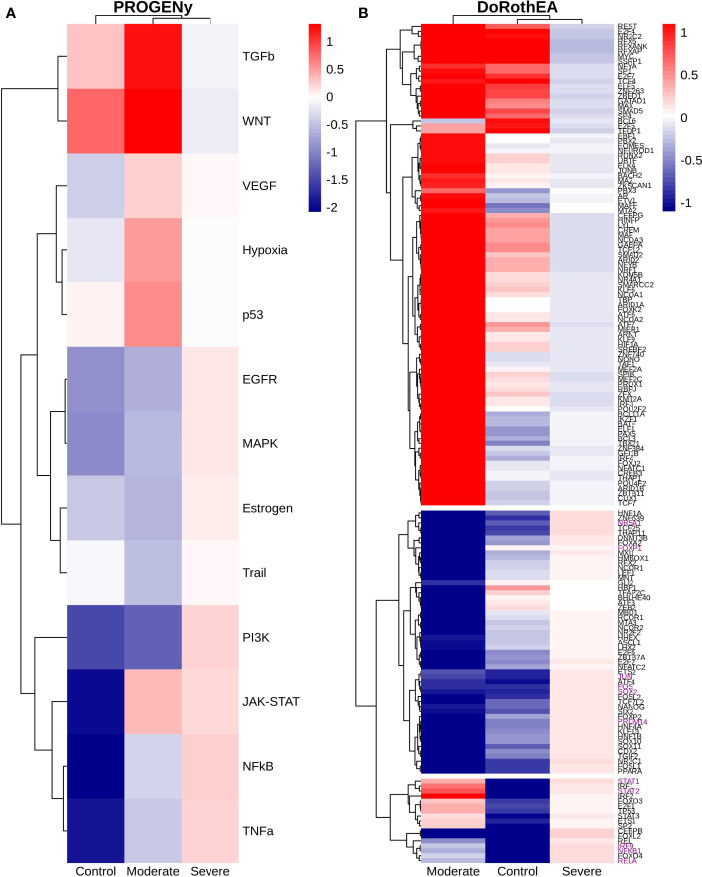
TFs associated with the disease severity in Dendritic cells. **(A)** Resulting Heatmap from DoROthEA analysis. **(B)** Resulting Heatmap from PROGENy analysis. Colorbar sets the activation (red) and inactivation (blue) values.

**Figure 9:**
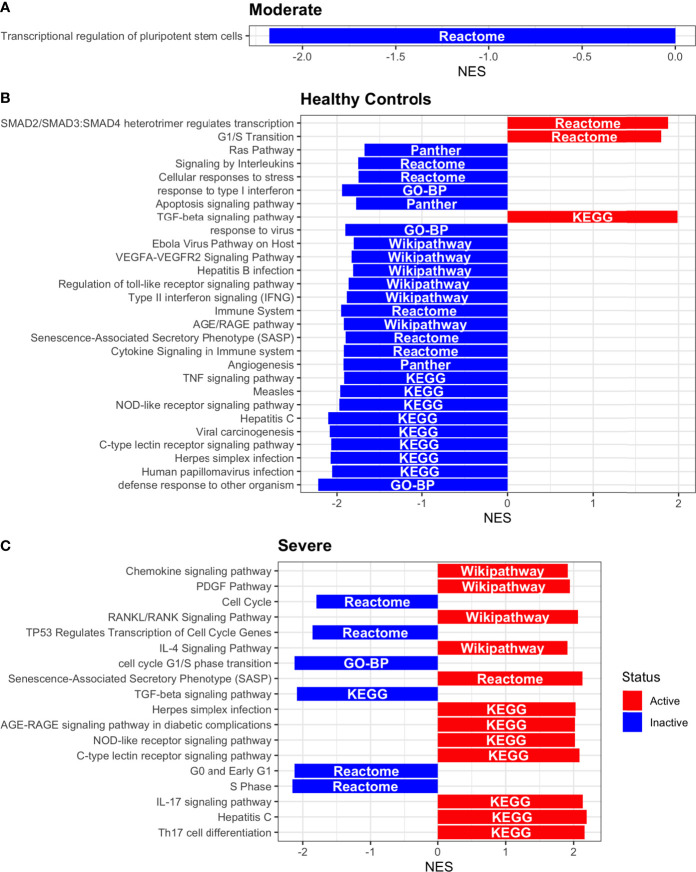
Pathway Enrichment Analysis across various databases. Activity status of the TFs in a particular pathway found by DoROthEA across the disease severity in Dendritic Cells. **(A)** GSEA in moderate patients. **(B)** GSEA in healthy controls. **(C)** GSEA in severe patients. The normalized enrichment score (NES) value represents the activity status within the disease severity conditions, a positive value for active pathways (red), and a negative value for inactive pathways (blue). The dataset for every pathway (rows) is indicated inside the colored bar.

Dendritic cells (DCs) are a class of bone marrow derived cells originating from lympho-myeloid hematopoiesis that link the innate sensing of pathogens and the activation of adaptive immunity. DCs recognizes and responds to pathogen-associated and danger-associated signals, molding the inflammatory response (132). To unveil how DCs diversify their functions according to the disease severity in COVID-19 patients, we inferred the TFs activities that guide the DCs phenotype using DoRothEA, as in previous sections (**Figure 8A**). Subsequently, to understand their functionality at a system level, we performed two complementary, yet independent, functional pathway analysis using PROGENy and gene-set enrichment analysis (GSEA) (**Figures 8B**, **9** and **Table S4**).

Having identified the single-cells classified as DC, we applied DoROthEA and obtained the TFs activated or inactivated within each disease severity. At a glance, our analysis showed that there was a clear signature dividing a set of active and inactive TFs in moderate patients (**Figure 8A**). However, for the severe patients and the healthy controls, the results indicate a more discrete activation or inactivation of some TFs included in DoRothEA database (**Figure 8A**). Through a carefully manual exploration of these TFs, we found that FOS, JUN, RELA, and NFKβ1 have an apparent differential behavior between the disease severity (**Figure 8A**). While severe patients activate these TFs, in moderate patients and healthy controls are inactivated. Notably, these TFs are associated with immune system pathways such as Th17 cell differentiation. This last pathway has, in turn, been related to a specific type of DCs, inflammatory DCs (infDCs) present in human inflammatory environments (133). This finding can be correlated to the disease severity suggesting that infDCs can be a potential driver in pathogenesis in COVID-19, as occurs in other inflammatory diseases (134, 135). Moreover, motivated by the marginal differences between the TFs of severe patients and healthy controls, we employed a gene-set enrichment analysis (GSEA) over the TFs list to systematically explore the potential phenotype of the DCs. Each enrichment plot represents a disease severity condition according to the active and inactive TFs (**Figure 9**). Briefly, we obtained one inactive pathway for the moderate patients and healthy controls, and severe patients collected active and inactive pathways. Specifically, the enrichment for the moderate patients showed the inactivation of the regulation of pluripotent phenotype, a result that is expected in a differentiated cell type (**Figure 9A**). Also, the enriched gene set possesses TFs such as FOXP1, NR5A1, PRDM14, and SOX2. By exploring the function of each of these transcription factors, we found that they include processes associated with the maturation and differentiation of dendritic cells (136, 137). To interpret the potential phenotype guided by the inactivation and activation of several TFs in the healthy controls, we identified that most inactive pathways are related to viral infection, immune response, and proinflammatory signaling. This condition is anticipated in cells that lack a viral infection (**Figure 9B**). On the other hand, the active pathways in healthy controls showed the cell cycle activation and TGFß signaling that function as pathways for maintaining DC homeostasis (138). Finally, for the severe patients, the resulting pathways from the GSEA using the TFs obtained by DoROthEA as input pointed that the DCs are implicated in four biological functions: 1) Inflammatory processes (RANKL/RANK signaling, AGE-RAGE signaling, NOD-like receptor pathway, C-type receptor pathway, Senescence Secretory Phenotype, and Il-17 pathway), 2) Differentiation and maturation (PDGF pathway, IL-4, and Th17 cell differentiation), 3) Response to viral infection (Herpes and Hepatitis B), and 4) homeostasis (cell cycle and TGFß pathway). The inflammatory processes, differentiation, maturation, and response to viral infection are activated while inactivating DC homeostasis.

In detail, the host innate immune system is the first line of defense after viral infection. Germline-encoded pattern-recognition receptors (PRRs) sense pathogen-associated molecular patterns (PAMPs) related to viral infection and are responsible for initiating the biochemical signaling cascades that orchestrate the innate immune response. PRRs are a large group of proteins that include either membrane-bound receptors or cytosolic receptors. Some examples of membrane-bound receptors include C-type lectin receptors (CLRs), and the cytosolic receptors compromise the nucleotide-binding oligomerization domain (NOD)-like receptors (NLRs) (139, 140). Both types of receptors are present in DCs, and their function is related to a particular subset of expressed receptors. For instance, the NLRs functions in DCs vary from the probable enhancement of T cell priming to the modification of TLR-induced maturation (141). This work presents evidence that both signaling pathways (NLRs and CLRs) are activated in severe patients. However, the exact receptor involved in the pathway cannot be pinpointed. The reason for this limitation is essentially in the DoRothEA database that only contains TFs. However, both pathways share a crucial activated core of TFs (IRF9, JUN, NFKB1, RELA, STAT1, and STAT2). In general, the resulting phenotype guided by the activation of both pathways promotes a pro-inflammatory environment in DCs (141, 142). In addition to the pathways mentioned above, other results strengthen the pro-inflammatory phenotype; for example, the RANK/RANKL pathway is involved in DCs inflammatory process (143). Additionally, the AGE-RAGE pathway induces the secretion of pro-inflammatory cytokines and does not lead to DCs maturation (144). Overall, the results indicate that the moderate patients and healthy controls possess several pathways involved in the maintenance of differentiation and homeostasis. In contrast, the severe patients show a pro-inflammatory program guided by several pathways and viral response, and indirect inhibition of maturation. Indeed, the obtained results in the severe patients match with multiple publications associating the SARS-CoV-2 infection with a pro-inflammatory environment. However, several questions remain open about why and how these pro-inflammatory pathways are not properly held to maintain the virus clearance?

Finally, the results obtained using PROGENy (**Figure 8B**) established an activation in several pathways. In the moderate patients, we observed activation in TGFß, WNT, VEGF, Hypoxia, p53, and JAK-STAT, while in the severe patients were activated the pathways EGFR, MAPK, Estrogen, PI3K, JAK-STAT, NFKß, and TNFα. By comparing these activated pathways that guide the DCs functions, we suggest a possible protective role in moderate patients, mainly because the activated pathways lead to a tolerogenic phenotype. In particular, the expression of TGFß in DCs can induce tolerogenic DCs (145). Tolerogenic DCs can mediate tolerance mainly by two events; one involves inducing anergy (inactivation of T cells), while the other induces apoptosis of antigen-specific autoreactive T cells (146). Also, tolerogenic DCs are characterized by an immature or semi-mature phenotype guided by the expression of low levels of costimulatory molecules, high secretion of anti-inflammatory cytokines, and decreased expression of pro-inflammatory cytokines (147, 148). This tolerogenic phenotype agrees with the obtained results in other activated regulons in the moderate patients, not only TGFß. For example, the activation of VEGF can reduce the differentiation of DCs (149), and the activation in the WNT pathway in DCs plays a significant role in regulating tolerance (150). In contrast, the severe patients show pathways associated with a higher maturation of DCs, for instance, the activation of NFKβ, which is crucial for the maturation of DCs (151), and the activation of TNFα, required to efficiently mature DCs during virus-mediated stimulation (152). Overall, it is clear that the pathways obtained by PROGENy control the homeostatic functions in DCs, such as migration, tolerance, antigen presentation, regulation of inflammation, immunosurveillance, maturation, and differentiation. However, the degree and the time of activation within the viral infection may lead to disparities in the severity. Here, we suggest that the tolerogenic DCs phenotype can lead to a less severe outcome, and the ineffective immune responses may be fundamental to unveil the severity within patients.

### 3.8 T Cells Role

T cells exert primary functions in viral containment and clearance. Their role on SARS-Cov-2 infection is negatively affected by lymphopenia, which is related to disease severity and majorly impacts these cells (61, 153–155). Our data analysis revealed massive recruitment of T cells in the alveolar compartment for moderate patients (**Figure 1C**). T cells promote the recruitment and activation of monocyte-derived inflammatory macrophages leading to a positive loop of immune overactivation and mass migration to the lungs, contributing to tissue damage (156, 157). Moreover, their role in the disease severity remains unknown.

**Figure 10:**
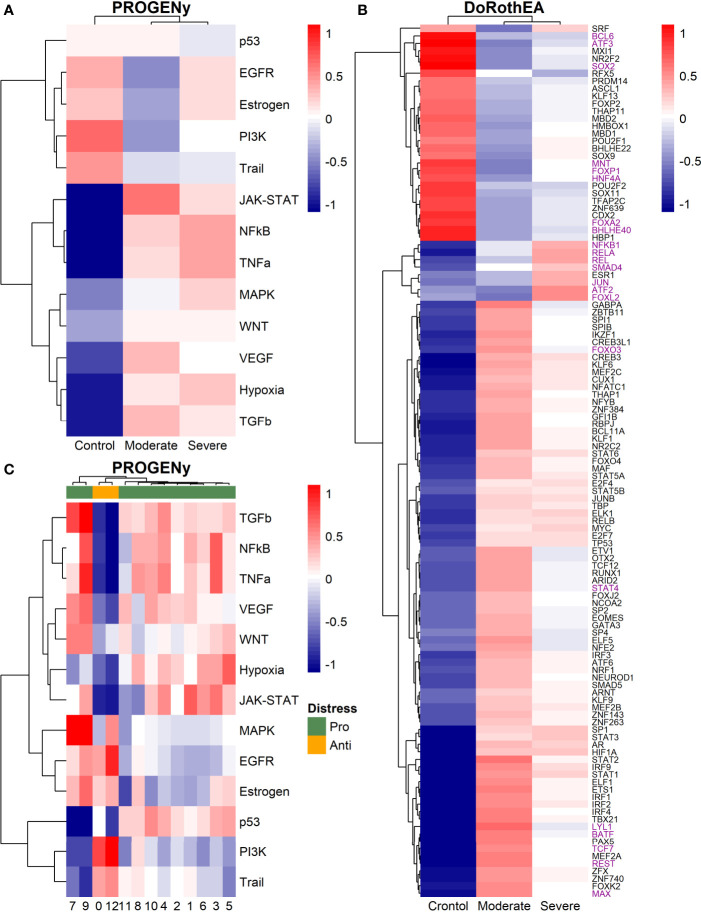
Dysregulated pathways and TFs in T cells. **(A)** Pathways activations results (PROGENy) according to health status. **(B)** TF results (DoROthEA) according to health status. Green square highlights those genes only activated on T-NK cells from severe patients. **(C)** Progeny analysis using mixing data of T and NK cells, green and orange groups are related to a pro and anti-distress response, respectively. Colorbar is related to activation (red) and inactivation (blue) values.

Functional pathways analysis with PROGENy showed pathway activation among patients and controls (**Figure 10A**). Furthermore, healthy controls inactivate pro-inflammatory pathways (Jak-Stat, NFkB, TNFα, VEGF, Hypoxia, and TGFβ). Thus, although results suggested that T cells from patients promote inflammation and cytokine release, each group of patients might do it differently. Along with the pro-inflammatory pathways activated for COVID-19 patients, the Jak-Stat pathway is the most activated, and it is related to moderate patients. Additionally, for this patient group, the VEGF and TGFβ pathways are overactivated. The Jak-Stat pathway is essential for the innate and adaptive antiviral response by the B, T, and NK cells; however, it promotes the cytokine storm (158). As far as the results are concerned, the Jak-Stat pathway is activated with the strongest response in T cells for moderate patients of all cell types, which might set one characteristic of a proper immune response. In the case of the overactivation of VEGF and TGFβ pathways, it seems counterintuitive in moderate patients. VEGF pathway increases vascular permeability and aggravation of endothelial damage, and TGBβ has been settled as a therapeutic target to improve COVID-19 prognosis (53). Severe patients have overactivated preponderant pathways (NFKβ, TNFα, and Hypoxia) to the pro-inflammatory phenotype. In concordance with previous reports (159), we observed that the T cells in the cytokine storm exacerbate lung immune response.

To identify relevant transcription factors orchestrating the pathways described above, we used DoRothEA analysis among samples (**Figure 10B**). We observed relevant genes in promoting viral clearance and long-lasting immune response based on the inactivated TFs on patients T cells (**Figure 10B**). For instance, BCL6 had inverse activation in healthy controls and patients. BCL6+ T follicular helper (TFH) cells interact with B cells in the germinal centers to produce antibodies and prompt long-lasting immune memory. Evidence supports that as COVID-19 aggravates, germinal centers and BCL6 expression are lost (160). ATF3 has an immunity role in Th2 and NK cells related to the negative regulation of pro-inflammatory cytokines (IL-4, IL-5, and IL-13) and INF-ℽ (161). MNT overactivation has an antagonist role to MYC (112, 113). MYC depletion sets Treg cells in quiescence, but its combination with PKC activates T and NK cells to produce pro-inflammatory cytokines (162). The activation of SOX2 and HNF4A also described in the NK behavior is related to mucus hyper-secretion (110, 111). RFX5 regulates antigen-presenting cell induction (163, 164). FOXP1 and FOXA2 regulate Treg cell suppressive function and inflammation (114, 115). Finally, BHLHE40 set a balance between pro-inflammatory and anti-inflammatory Th1 cell fate determination inducing INF-ℽ expression, an innate immune response to viral infections (165, 166). However, the activation of INF-ℽ is considered adjuvant to cytokine storm (36, 167). Despite the results suggesting a non-responsive state for the patients, it has to be considered the temporality of the response. T cells from moderate patients trigger an initial solid response that decreases over time, whereas severe patients might induce a poor or null response (168). Besides, we found a collection of transcriptional factors only activated by the severe group (**Figure 10B**). Among them, we found subunits of the complex NFKβ: NFKB1, RELA, and REL. RELA is one of the most important transcription factors regulating the response to COVID-19 (169). All of them are involved in the NFκB pathway activation controlling the inflammatory response. The NFkB pathway has been proposed as a therapeutic target to treat acute infection in severe patients as it regulates cytokines liberation (107). The transduction protein SMAD4 acts as a partner to facilitate the translocation of RSmads into the nucleus modulating profibrotic genes (170). Another TF related to the profibrotic response is JUN, an *in silico* study associates JUN as a therapeutic target to reduce fibroblast proliferation and inflammation (170, 171). ATF2 regulates the expression of JUN through homo-dimerization or hetero-dimerization (172), consequently, might control pulmonary fibrosis. Finally, FOXL2 is related to T cell activation in ovarian cancer and promoting apoptosis (173, 174).

As for the severe patients, there are TFs only activated for the moderate patients that set the possible mechanism differentiating the T cells response as the disease aggravates. We considered the slight overactivation in severe patients of some TF as non-activated due to their values close to zero. For instance, FOXO3 deficiency induces inadequate T cells immune response by promoting the expression of IL-6 and NFKβ (175). STAT and IRF genes regulate the JAK-STAT pathway involved in the inflammation process. LYL1 has a pivotal function; overexpression of this TF induces poorly mature T cells, and its deficiency limits the clonal expansion necessary for a proper response (176, 177). BATF controls the production of Th17 cells, which in turn coordinates the pro-inflammatory response. In COVID-19 Th17 cells have been identified as therapeutic targets to regulate the cytokine storm (178). TCF7 is essential for T cell development and differentiation through promoting T cells differentiating to Th2 (memory T cells) fundamental in the immune response (179). REST is induced under hypoxia and regulates several hypoxia-repressed genes (180). Another regulator in the T cell differentiation is MAX. MAX forms a heterodimer with MYC tuning cellular growth. Our results suggest an intricate responsive state in the T cells associated with moderate patients. Nevertheless, previously mentioned mechanisms might be off in severe patients, indicating possible non-responsive T cells to the virus.

We evaluated the response of T cells according to the disease severity. Moreover, the activated pathways may show a hidden activation pattern due to the heterogeneity in the lymphocyte specialization. To evaluate cell heterogeneity and relate a particular function of the severe data, we mixed the T cells data from all healthy controls and patients and repeated the clustering and progeny analyses (**Figure 10C**). Given these new clusters and their activated pathways, we classified the T and NK data into two groups based on the expression profile and the dendrogram: pro-distress (green clusters: 7, 9, 11, 8, 10, 4, 2, 1, 6, 3, and 5) and anti-distress (yellow clusters: 0, and 12). We defined distress as the condition that promotes acute respiratory syndrome in COVID-19 infection. The pro-distress group had activated pathways that promote inflammation, increase tissue permeability, boost fibrotic response, hypoxia, and cytokine release (NFKβ, TNFα, JAK-STAT, TGFβ, EGFR, VEGF, and Hypoxia). Cluster 9 had the highest activation of the described pro-distress pathways, mainly conformed by severe patient cells. Moreover, there is an opposite activation between the pro and the anti-distress groups, showing modularity in the disease development. Thus, despite evidence showing T cells aggravate the disease severity, there is a subpopulation of cells that do not promote inflammation and cytokine liberation. Therefore, data suggested that even severe patients had T cells potentially helpful to fight back against the disease.

### 3.9 B-Cells Function Regulation According to Disease Severity

**Figure 11:**
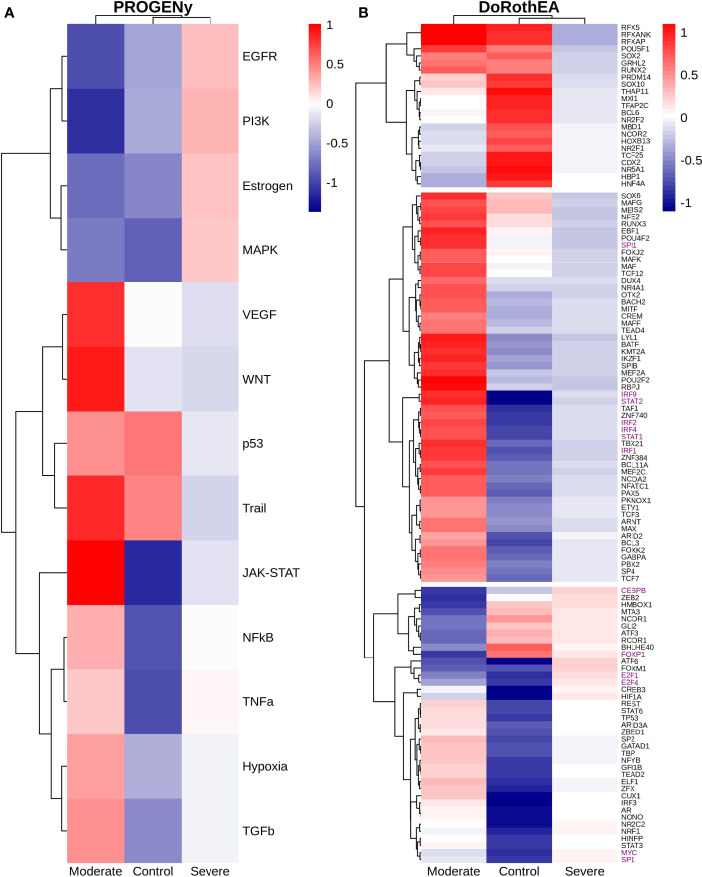
TFs associated with the disease severity in B cells. **(A)** Resulting Heatmap from DoROthEA analysis. **(B)** Resulting Heatmap from PROGENy analysis. Colorbar sets the activation (red) and inactivation (blue) values.

**Figure 12:**
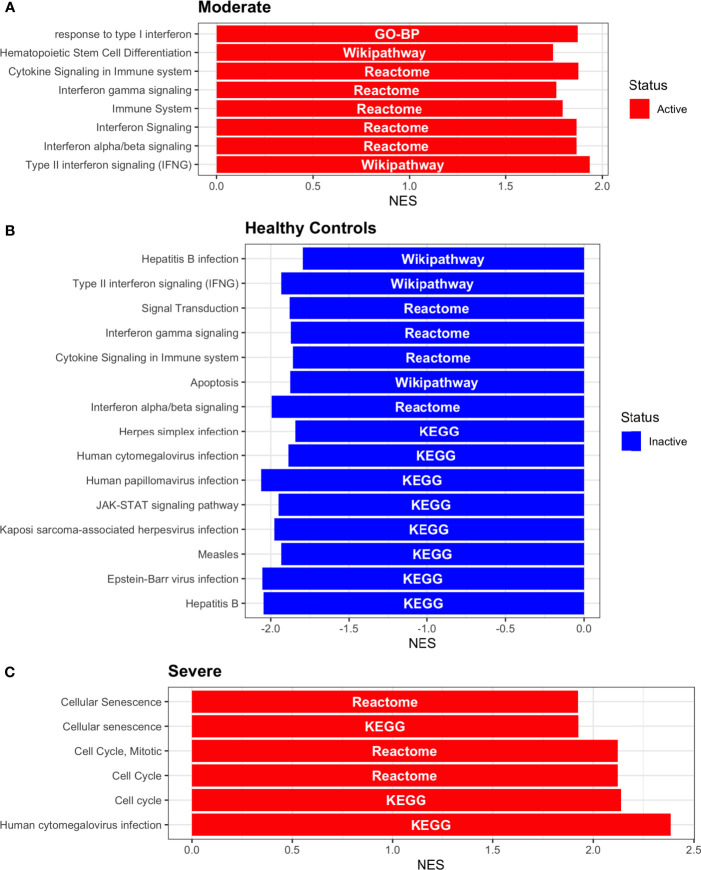
Pathway Enrichment Analysis across various databases. Activity status of the TFs in a particular pathway found by DoROthEA across the disease severity in B-cells. **(A)** GSEA in moderate patients. **(B)** GSEA in healthy controls. **(C)** GSEA in severe patients. The normalized enrichment score (NES) value represents the activity status within the disease severity conditions, a positive value for active pathways (red), and a negative value for inactive pathways (blue). The dataset for every pathway (rows) is indicated inside the colored bar.

B-cells are well known for their ability to produce antibodies. However, B-cells also perform several immunological functions, including antigen presentation, the production of multiple cytokines, and restrain excessive inflammatory responses (181). Here, we explored the phenotypic capacity of B-cells according to the disease severity in COVID-19 patients following three steps. First, we inferred the TFs activities that guide the B-cells phenotype using DoRothEA (**Figure 11A**). Second, we implement a functional pathway analysis using PROGENy (**Figure 11B**). Besides, we complemented these studies by accomplishing GSEA with several databases over the TF list in each disease severity condition (**Figure 12** and **Table S4**). From DoROthEA results, we obtained the TFs that are activated or inactivated within the disease severity. In general, moderate patients highly activate IRF1, IRF2, IRF4, IRF9, STAT1, STAT2, and SPI1(**Figure 11A**). Intriguingly, these TFs mediate the activation of both innate and adaptive immune responses, growth, and differentiation of B-cells primarily by regulating interferons (IFNs). Thus, in moderate patients, it is more likely that B-cells contribute to antiviral functions by inducing the transcription of ISGs (IFN-stimulated genes), which restrain unique stages of viral replication. These findings agree with other reports, showing a robust IFN-I response in a moderate infection (182). Moreover,severe patients activate E2F1, E2F4, MYC, FOXP1, CEBPB, and SP1 (**Figure 11A**). These TFs are primarily associated with cell cycle control, B-cell development, differentiation, and immune responses. In this context, previous publications showed that viral infection of human B cells can result in the activation of the cell cycle directly or indirectly by promoting the related cell signaling (183, 184). However, the precise effect in the cell cycle guided by SARS-CoV-2 is still unknown. To give more detail in the overactivation of the cell cycle effect in severe COVID-19 patients, we further discussed these results by employing a complementary approach such as GSEA. In healthy controls, most TFs are activated in moderate and severe patients are inactivated (**Figure 11A**). Notably, this TFs pattern indicates the futility of activating pathways involved in antiviral responses in a condition that lacks a viral infection.

Additionally, as we mentioned earlier, we employed a GSEA over the list of TFs identified with DoRothEA to explore the potential phenotype of the B-cells. Results for the moderate patients showed activation of pathways involved in the interferon response and the activation of cell differentiation (**Figure 12A**). Besides, in healthy controls, we obtained inactivated pathways related to viral responses (herpes, human papillomavirus, cytomegalovirus, hepatitis B, measles, Epstein-Barr virus; **Figure 12B**). In contrast, the results in severe patients showed activation of pathways involved in the cell cycle control, virus infection, and cellular senescence (**Figure 12C**). Especially by comparing the infected conditions, the IFNs response is activated in moderate patients and absent in severe patients, which can be associated with the clinical outcome. As mentioned in previous sections, the IFNs response (principally type I IFNs) functions as immunoregulatory cytokines that play a pivotal role in viral immunity and promote B cells survival, activation, and differentiation (185). The fact that type I IFNs have been reported to improve B cells survival (186) can explain in part the higher abundances of B cells in moderate patients compared to severe patients (**Figure 1C**). Also, in severe patients, the activation of cellular senescence can be explained by contributing to an inflammaging phenotype. These results correlate with the age variable in the severe samples, mainly being above 62 years old. In detail, inflammaging increases low-grade chronic inflammation related to age; a significant driver of this phenotype is cellular senescence (187, 188). In addition to these findings, other reports present evidence that the senescent B-cells subset is highly inflammatory with low proliferative capacity (189). Overall, B-cell cytokine production guided by IFNs response contributes to virus clearance in moderate patients. In contrast, in severe patients the B-cell senescence activation is partially responsible for inflammatory state. ​​Hence, we can conclude that B-cells can orchestrate the restrain of the viral infection by activating the type I IFN response in moderate patients. However, the potential B-cells senescent state in severe patients can drive the hyper-inflammatory environment correlated with a worse outcome in COVID-19 patients.

Finally, the results obtained using PROGENy (**Figure 11B**) supported an activation in several pathways unnoticed in GSEA. For instance, in the moderate patients, we observed activation in VEGF, WNT, p53, Trail, JAK-STAT, NFKβ, TNFα, Hypoxia, TGFß while in the severe patients EGFR, PI3K, Estrogen, and MAPK are activated. By comparing these sets of activated pathways that guide the B cells functions, we can explain in part the severity observed in patients, mainly because the activated pathways in the moderate patients lead to pro-apoptotic phenotype. In particular, the expression of Trail in B cells can induce preferential apoptosis for CD5(+) B cells (190), and p53 prevents the accumulation of progenitor B cells, thereby reducing the likelihood of incorrectly differentiated B cells (191). Also, the moderate patients show pathways associated with a higher maturation of B cells. For instance, NFKβ and TNFα signaling need to be active under a nonstimulated environment and during virus-mediated stimulation (151, 152). In addition to these results, the JAK-STAT signaling pathway, specifically Stat3 and Jak1, is involved in B cells developmental responses (192), and TGFβ1 is a potent regulator of B cell development from the pre-B cell stage up to immunoglobulin-secreting plasma cells (193). In contrast, the severe patients show pathways associated with B-cells identity, activation, proliferation, and survival guided by PI3K, Estrogen, and MAPK pathways (194–196). However, in the particular case of the EGFR pathway, other reports demonstrate that it promotes viral replication through increased virion uptake or suppression of cytokine production (197) This specific case only applies when the virus can infect B-cells. Moreover, EGFR appears to play a significant role in the severity of non-lethal infections (Influenza A) such that when it is inhibited, the disease is more severe. However, in the highly lethal infections (H5N1 or SARS-CoV), other mechanisms potentially cloak the role of EGFR (198). In general, the pathways obtained by PROGENy suggest control of the homeostatic functions in B cells, such as tolerance, development, maturation, and proliferation. In severe patients, we observed a higher activation, proliferation, survival, and infection pathways of B-cells. However, two questions remain open: Do the pathways involved in proliferation, and survival possess defects that jeopardize the B-cells function?, and Does the activation of EGFR, PI3K, MAPK, and Estrogen pathways trigger chronic inflammation?. Thus, a thorough understanding of how B-cell proliferation and survival are regulated could provide new insights for different severity conditions in COVID-19.

### 3.10 Neutrophils Function Regulation According to Disease Severity

**Figure 13:**
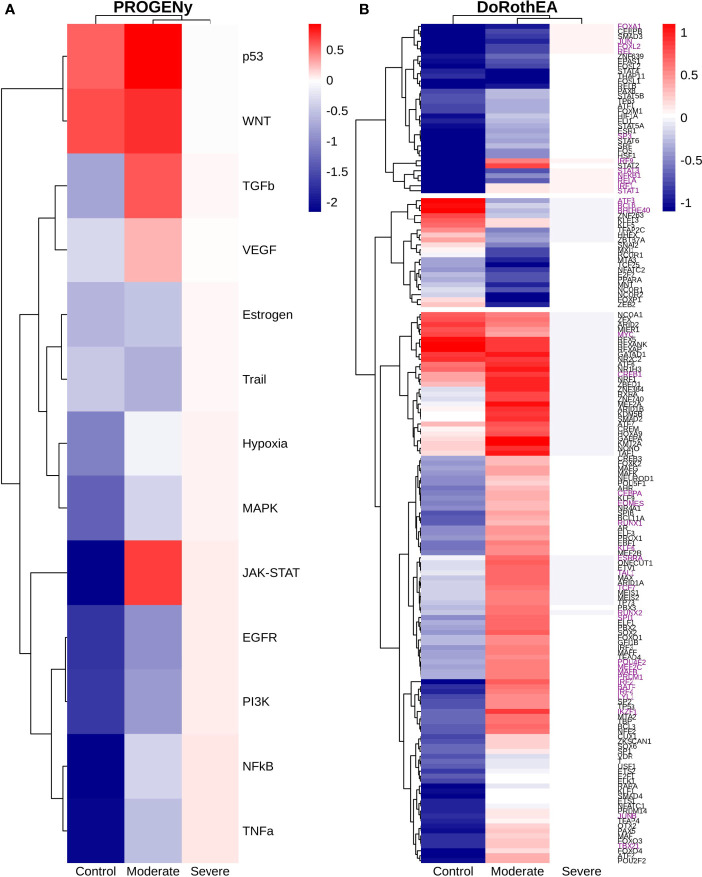
TFs associated with the disease severity in neutrophils. **(A)** Resulting Heatmap from DoROthEA analysis. **(B)** Resulting Heatmap from PROGENy analysis. Colorbar sets the activation (red) and inactivation (blue) values.

**Figure 14:**
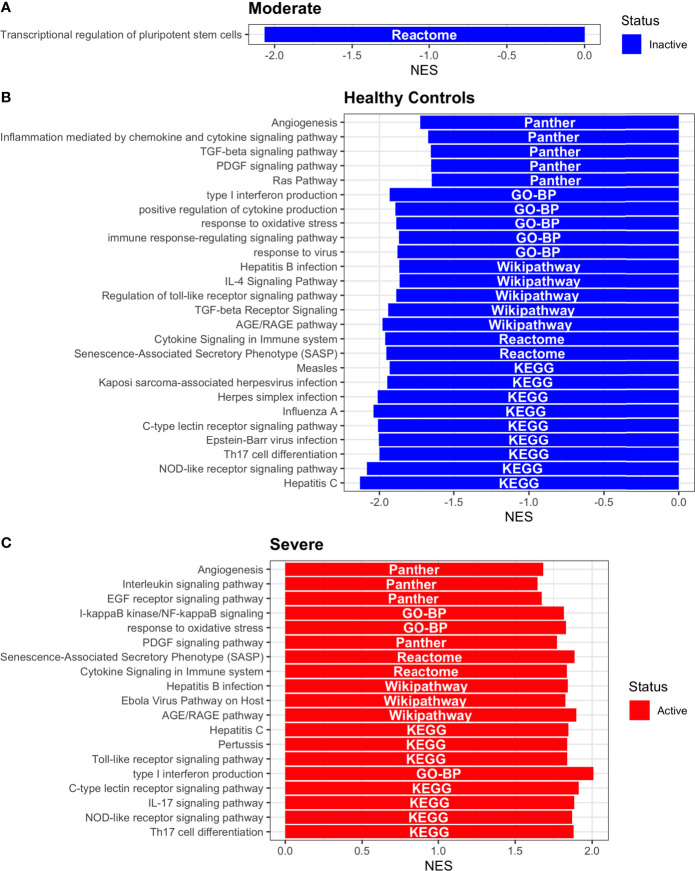
Pathway Enrichment Analysis across various databases. Activity status of the TFs in a particular pathway found by DoROthEA across the disease severity in Neutrophils. **(A)** GSEA in moderate patients. **(B)** GSEA in healthy controls. **(C)** GSEA in severe patients. The normalized enrichment score (NES) value represents the activity status within the disease severity conditions, a positive value for active pathways (red), and a negative value for inactive pathways (blue). The dataset for every pathway (rows) is indicated inside the colored bar.

Neutrophils are the most abundant immune cells in human blood. They are present in many lung diseases associated with acute respiratory distress syndrome (ARDS), as described in infections led by the influenza virus and SARS-CoV-1 (199). Even though their particular role during viral infection is not fully understood. Several reports suggested that neutrophils enhance antiviral defenses by interacting with other immune cells subpopulations, promote cytokine release, oxidative burst, neutrophil extracellular traps (NETs), degranulation, virus internalization, and virus clearance mechanisms (200, 201). This section analyzed the potential phenotypes of neutrophils corresponding to the disease severity in COVID-19 patients. As described in previous sections, we employ a three-step analysis conducted by the inference in the TFs activities using DoRothEA (**Figure 13A**). Consecutively, we implement two independent functional pathway analyses, on the one hand, PROGENy (**Figure 13B**) and on the other hand GSEA for each disease severity condition (**Figure 14** and **Table S4**). From DoROthEA results, we obtained the TFs that are activated or inactivated in each disease severity condition. In general, moderate patients highly activate an extensive list of TFs involved in the leukocyte differentiation (BATF, CEBPA, CEBPB, CREB1, EOMES, ESRRA, IKZF1, IRF1, IRF4, JUNB, KLF6, LYL1, MAFB, MEF2C, MYC, POU4F2, PRDM1, TBX21, RUNX1, RUNX2, SPI1, TAL1, and TCF7; **Figure 11A**). Also, the results showed the activation of GABPA, NRF1, and CREB1, which in turn participate in the neutrophils maturation, modulate oxidative stress response, and cytokine production, respectively (202–204). Thus, in moderate patients, most activated functions are implicated in maturation, differentiation, and response to stress stimulus. Moreover, severe patients showed a slight activation of some TFs: STAT3, STAT1, RELA, JUN, NFKB1, REL, FOXL2, SP3, FOXA1, and IRF1 (**Figure 13A**). These TFs are primarily associated with the mediation and stimulation of pro-inflammatory cascades (205, 206). To present a particular function in the slightly activated pro-inflammatory cascades in severe COVID-19 patients, we further discussed these results by employing a complementary approach such as GSEA. In healthy controls, most inactivated TFs are related to virus response (FOSL1, IRF1, IRF2, IRF9, RELA, STAT1, STAT2; **Figure 11A**). Besides activated TFs in healthy controls such as ATF3, BHLHE40 and BCL6 are modulators of neutrophil inflammation (73, 207, 208) (**Figure 13A**). Notably, in healthy controls, the TFs pattern indicates the lack of response to external stimuli, particularly a virus infection, while activating the inhibition of inflammation.

Furthermore, we employed a GSEA over the list of TFs identified with DoRothEA to survey the potential phenotype of the neutrophils. Results for the moderate patients present an inactivation of a unique pathway involved in regulating pluripotent phenotype. This result was also found in DCs and may explain the same aspect of reducing this biological program in a differentiated cell type (**Figure 14A**). Besides, in healthy controls, we obtained inactivated pathways related to viral responses (herpes, hepatitis B, hepatitis C, measles, Epstein-Barr virus), antiviral pathways (IFN-1 production), and inflammatory-related pathways (C-type lectin receptor signaling pathway, NOD-like receptor signaling pathway, Th17 cell differentiation, and inflammation guided by chemokine and cytokine signaling; **Figure 14B**). In comparison, the results in severe patients showed activation of pathways involved in the cellular senescence, angiogenesis, and several pro-inflammatory pathways (**Figure 14C**). Intriguingly, as the results in DCs, the comparison in the activation status between infected conditions showed that while the neutrophils functions in moderate patients led to a pronounced maturation, differentiation, and response to stress stimulus. Thus, processes in severe patients are related to a pro-inflammatory phenotype. However, severe patients might induce angiogenesis. Neutrophils can stimulate angiogenesis *via* matrix metalloproteinase-9 (MMP-9) due to herpes simplex infection (209). Nonetheless, the protective or harmful effects guided by the activation of angiogenesis in SAR-CoV-2 disease are still unknown. In this regard, the pronounced pro-inflammatory phenotype observed in severe patients can also play a detrimental role. Another report demonstrated that an excessive neutrophil activation linked to a pro-inflammatory transcriptional signature correlates with lethal influenza infection (206). Also, through prolonged or excessive responses in the production of pro-inflammatory cytokines, neutrophils can cause extracellular matrix damage, extensive cell death, and tissue necrosis (201). Together these reports suggest that neutrophils (as other cell types) pro-inflammatory pathways can drive the hyper-inflammatory environment that corresponds to a worse outcome in COVID-19 patients. However, from our results, a question remains open: Is the slight activation of pro-inflammatory pathways observed in severe patients sufficient to promote a hyperinflammatory environment?

Finally, the results obtained using PROGENy (**Figure 14B**) established an activation in several pathways. We observed activation in p53, WNT, TGFß, VEGF, and JAK-STAT in the moderate patients, while in the severe patients MAPK, JAK-STAT, NFKß, TNF-α, EGFR, and PI3K were slightly activated. By comparing these activated pathways that guide the neutrophils functions. In particular, the expression of p53 in neutrophils can induce apoptosis (210). The determination of the lifespan in neutrophils can be controversial due to the beneficial or detrimental effects (211). Some evidence indicates that inflammatory signals prolonged neutrophil survival in bacterial infections (212), and NFKß and PI3K activation delay neutrophil apoptosis in a mimic viral infection (213). Moreover, neutrophil apoptosis is inhibited in human cytomegalovirus infection(HCMV) (214), while simian immunodeficiency virus (SIV) induces neutrophil apoptosis, and the extent of apoptosis correlated positively with disease severity. Thus in some infections, apoptosis shows both a protective and detrimental role. Here, we can infer that the plausible induction of apoptosis by P53 can lead to a protective role in moderate patients. Additionally, in moderate patients, the functions related to the activation of TGFß, VEGF and JAK-STAT can be the induction of chemotaxis, inflammatory angiogenesis, and pro-inflammatory signaling, respectively (215–217). In contrast, the severe patients show the slight activation in pathways associated with a pro-inflammatory phenotype, for instance, the activation of JAK-STAT (217), TNF-α, required in the release of NETs (218), and VEGF, which contribute to neutrophilic inflammation through enhanced production of IL-8 (219). Overall, it is clear that the pathways obtained by PROGENy control the homeostatic functions in neutrophils, such as apoptosis, chemotaxis, angiogenesis, and pro-inflammatory signaling. However, in both disease conditions, pro-inflammatory signaling is present, with higher activation in moderate patients. These results seem contradictory since the worse outcomes are prevalent in hyper-inflammatory environments. Nonetheless, in moderate patients, the complementary pathways related to apoptosis can adequately remove the neutrophils population to restrain the potential reactive effects. Likewise, in severe patients, the possible slight promotion of NETs by TNF-α can drive the hazardous results observed in COVID-19 patients (220). Here, we suggest that the activating neutrophils apoptosis can efficiently modulate inflammatory phenotype in moderate patients, and the robust yet slight inflammatory phenotype in severe patients may be sufficient to drive a worse outcome.

## 4 Discussion

The COVID-19 pandemic has driven a sanitary emergency that has caused millions of deaths worldwide. Patients have been treated and classified according to the severity of their symptoms (221). Moreover, biomarkers are used clinically for many conditions reflecting pathological development. Particularly for COVID-19, the proposed severity markers are isolated and have not been analyzed integrally (222). Additionally, although the changes that undergo some immune cells have been described, there is a lack of information about how the different immune cells promote or restrain the disease on the whole. Using BALF single-cell data from 3 healthy controls and 9 patients classified as moderate and severe (1), we focused this study on two issues, illness severity classification and the lack of description regarding immune cells as a whole. Hence, first, we proposed using a classifier to differentiate moderate and severe patients according to their single-cell gene expression profile. Second, we characterized the immune hallmarks based on the activation of the identified immune cells.

Using our XGBoost classifier, we obtained high accuracy (0.98) in distinguishing moderate and severe patients samples (**Figure 2**), an improved approach compared with similar models in other studies with different human samples (223, 224). Furthermore, we evaluated the model applicability in another BALF data (21) with a high rate of accuracy. Thus, by getting a severity COVID-19 gene-signature, we proposed a precise alternative method to classify disease severity. Moreover, we are aware that this result needs to be proved in more extensive datasets from across the globe, which sets future work. In addition, we identified a genetic signature conforme by 8 genes and participating in various functions. Congruent with previous reports (225, 226), this diversification of genes can be related to the systemic damage due to the SARS-COV-2. Moreover, to accurately describe the systemic dysregulation among patients, it is necessary to evaluate the SARS-COV-2 effect in each cell type involved in the infection dissemination.

The SARS-CoV-2 has a primary entrance to the human body through respiratory epithelial cells. Moreover, the virus also infects and impairs monocytes and macrophages (227, 228, 230). The axis monocytes-macrophages-NK-DCs coordinate the innate immune response and promote viral clearance. Still, as a consequence of SARS-CoV-2 infection, they get dysregulated, promoting excessive cytokine release affecting adaptive immunity (32, 64, 228, 229, 231, 232). We described this downhill effect in three main dysregulated processes in which the found cells take part: dysregulation of the innate and adaptive immune response and the cytokine mayhem.

The strike of innate immune response starts with the pulmonary epithelial cells. They control the balance between limiting the viral spread and causing excessive inflammation and tissue destruction by cytotoxic immune cells (233). Results showed that AT1, AT2, and secretory epithelial cells from severe patients had activated STAT and IRF genes, suggesting IFN-I signaling is on for them and off for moderate patients. A relevant consideration is that almost all patients (except for one moderate) were treated with interferon. For severe patients, samples were collected 6 to 10 days after initiation of the treatment; for moderate patients were collected 2 to 3 days after. An *in-vitro* study showed that IFN signaling was time-dependent, it takes time to ISG, and IFN signaling mediated by TGFβ developed an antiviral state (234). Therefore, we hypothesize that both patient groups were affected by the virus, but the severe ones had the manifestations of the pharmacological effect. Moreover, STAT1 is associated with the cytokine storm (34), congruently with our findings. Additionally, we observed an activity enhancement of several TFs leading to possible tissue fibrosis for the severe patients through the impairment of TGFβ, inflammation, activation of EMT, and EndMT pathways (**Figure 3C**) effect that we proposed is led by the AT1, AT2, and secretory epithelial cells (**Figure 4D**). These effects heighten the fact that despite ciliated, and squamous cells have higher infectivity rate and ACE2 expression by the SARS2-CoC-2 than AT1 and AT2 (235), alveolar epithelial cells seem to be developing the infections havocs modifying their initial innate response. Innate immune response promotes the recruitment of the immune cells, activates the antigen presentation process, and activates pathogen clearance. After the epithelial cells get infected, monocytes, macrophages, and NK are the firsts cells to be recruited.

Monocytes and macrophages are two fundamental immune cells with pivotal functions to induce immunity and host defense against foreign agents. Although these cells have been reported to promote COVID-19 infection in severe patients (236), the origin of this behavior remains an open question. Moreover, we postulate how these cells interact between them. According to our cell classification, there is no difference between monocytes and macrophages proportions for the patients (**Figure 1C**). Quantification of HLA and IFN-stimulated genes (IFI6) correlated with pathogen detection, and the inflammatory response showed high expression in monocytes of moderate patients (**Figure 5**). This critical condition for severe patients may lead to a deficient or null peptide antigens presentation to the immune system that modulates the production of antibodies against foreign peptide antigens (61, 153–155). Moreover, this inadequate antigen production is driven by IL-6, usually found in high concentrations in severe patients (61, 228). Additionally, monocytes from moderate patients suggest a controlled inflammatory response is fundamental for viral clearance related to IFI6 expression. Congruent with reports, the data showed insights into the already reported non-regulated inflammatory landscape and immune evasion by the immune response in severe patients associated with SARS and systemic failure (225, 226, 237). The immune system cells may get involved in several critical changes within disease severity. It has been suggested that monocyte-macrophage activation can induce subsequent respiratory failure in severe patients (156). Macrophages from severe patients activated genes and pathways related to IL-6 driven inflammation and cytokines release such as TNFα pathway, MAPK pathway, the axis TREM2-APOE-LGALS3, and the antigen SMAD family genes; to name a few (**Figure 6**). Thus, in severe patients, monocytes/macrophages guide the innate immune response to a dangerous positive inflammation loop where monocytes and macrophages induce IL-6 secretion, which hit antigen presentation, promote IL-6 pathway activation, and pro-inflammatory cytokines release, promoting innate cells recruitment so on. This chicken-and-egg condition hypothesizes that the breakpoint into this loop is the timing of the first immune response and is still an open question to unravel COVID-19 dynamics.

NK cells complement the innate immunity directly attacking foreign pathogens. Thus, their immune role has a balance between protection against pathogens and preventing autoimmune responses. Moderate patients seem to induce an effective immune response by the lysis of target cells through cytotoxic degranulation and controlling an auto-apoptotic response. NK cells regulate Th cells; for severe patients, this regulation impairs the adaptive immune response. The NFKβ pathway seems to be playing a significant role for these cells and controlling inflammation for severe patients.

The description of the immune response continues with the DCs as they are the link between innate and adaptive immunity. The primary function of adaptive cells is to mediate the polarization of innate cells into effector cells and antibody production (238). Interestingly, DCs have similar behavior as B cells and neutrophils. According to our data, the main characteristic was observed: whereas the moderate group has mature and differentiated DCs and neutrophils, severe patients show a homeostasis dysregulation promoting cytokine release and inflammation (**Figures 8**, **9**, **13**, **14**). Particularly for DCs, there might be a negative effect mediated by the Th17 immune response (**Figures 8**, **9**). Th17 cells differentiate in part through the actions of IL-6 (239). IL-6 induced by coronaviruses in the lung appears to promote in susceptible hosts Th17 responses that may lead to severe lung pathology (240). In addition, neutrophils and DCs from moderate patients seem to be regulated by a tolerogenic effect setting an equilibrium point that controls the pro-inflammatory response. As for the severe patients, immature and proliferative cells lead to a possible non-development adaptive response making them susceptible to enter into the positive inflammation loop. Therefore, B and DCs from severe cases might have a delayed response and senescence state promoting viral replication and make the immune response susceptible to enter into the positive inflammation loop. Particularly for B and neutrophils, cellular senescence and the activation p53 pathway induce the pro-inflammatory state. As an important remark, DCs, B, and neutrophils from moderate patients strongly activate inflammatory regulation pathways, but severe patients show a slight activation of pro-inflammatory pathways. This condition suggests that pro-inflammatory pathways do not need an exacerbate activation to induce a magnified production of cytokines.

As the adaptive immune response is triggered, the role of T cells emerges. Data from patients showed activation of pro-inflammatory pathways. Although T cells from patients have inactivation of BCL6, ATF3, and MNT affecting immune memory formation and B cells maturation, moderate patients B cells showed activated pathways suggesting an adequate antiviral response. An effect that cannot happen with impaired B cells. Moreover, an important consideration is the dynamics; T cells in moderate patients have non-stationary dynamics that decrease after reaching their peak concentration (168, 241). According to this hypothesis, moderate patients activated TFs that regulate the expression of IL-6, NFKβ, and Th17 suggesting a controlled inflammatory state contrary to severe patients. Interestingly, although severe cells promote inflammation by the activation of NFKβ, not all cells take part. Some of them potentially may induce an anti-distress condition. Moreover, the pro-distress is so strong that only a few T cells are needed to dysregulate the system and push it into the positive inflammation loop to end a systemic failure.

On the whole, used data might reflect an advanced stage in the infection. At this stage, moderate patients exert a possible efficient immune response settled by an IFN-I decrease and the presence of pro-inflammatory cytokines setting the precise balance promoting viral clearance and immune memory. Furthermore, the immune response involves several activation/inactivation from epithelial response through mature B cells. On the contrary, severe patients might be reflecting their dysregulation starting with some innate immunity cells (monocytes, monocytes, and NK) promoting IL-6 and innate cell recruitment, which exert a massive pro-inflammatory condition affecting the adaptive response blocking antigen presentation. Carrying on this downfall process, immature DCs impairment induces massive cytokine release, leading to more innate cell recruitment, cytokine release, and fibrosis. T, B, DCs, and neutrophils exacerbate the condition releasing IL-6 deploying an infective adaptive response aggravated by the loss of germinal centers, consequently impairing immune memory. Thus, despite severe patients showing activation IFN-I, their response might not be adequate. NFKβ pathway has been identified impaired in the broncho-alveolar space cells; mostly all the analyzed cells show impairments. Overall, severe patients activated the described cells pushing the system into a hyper-inflammation state that might cause patients to die.

Several studies have used the same dataset to evaluate the immunological effect in COVID patients. Among the differences: Park and Lee identified the effect on neutrophils glucocorticoid receptors on severe patients and described in general terms cytokines released by myeloid cells (15). Overholt el al. studied balf and pbmc samples finding differences among compartments setting the crosstalk among them (242). Garg et al. perform a meta-analysis with balf data and pbmc. They studied myeloid impairment independently for each cell (243). Finally, Tang et al. analyzed the origin of cytokine release on balf and pbmc samples (244). Moreover, all the previous studies had different perspectives correlating the airway and blood compartments, and centered their study in one cell-type. As their valuable endeavors, they do not describe a systemic interaction among cells and how the downhill effects are transduced to the other cell-types comprising patients to a dreadful or hopeful condition. In addition, we implemented a decision model useful to classify patients.

It is essential to consider that patients were under pharmacological treatment at the moment samples were taken. Four of six severe patients took interferon, methylprednisolone, and ribavirin. Methylprednisolone is an immunosuppressive drug; it stops or delays pneumonia. Moreover, severe patients did not show an immunosuppressive effect. Cells for this group have activation of pro-inflammatory pathways and cytokine release. Therefore, the drug effectiveness might be reconsidered. All patients except one moderate were under interferon treatment. Despite the implication that the interferon might be beneficial, we considered that these effects are conserved across data, excluding a possible bias.

As a final remark, the present study set the integration of cells belonging to the immune landscape for COVID-19 patients. Having each cell’s global and particular contribution made the therapeutic options clearer with their scopes and limitations. Along with this work, we have drawn some future perspectives. First, as soon as more available single-cell RNAseq data from BALF and PBMC, it will allow us to explore the scope of our conclusions and its limitations regarding variables such as ancestry, treatments, and the prevalence of different comorbidities. Second, given the complex nature of the disease, we should move toward integrative studies capable of integrating clinical data and other high-throughput technologies that complement our conclusions and set tactics to control illness severity. Machine-learning and systems biology are indispensable tools to reach this goal (245, 246). We expect this integrative strategy to elucidate how COVID-19 affects our health and eventually design suitable strategies to reduce their lethal consequences in human life.

## Data Availability Statement

The scRNA-seq data used in this study is publicly available in GEO (GSE145926) (1). The criteria of cellular classification can be found in **Table S1**. All the code is available at https://github.com/resendislab/Covid-19_scRNAseq.

## Author Contributions

AV-J, UA-PDL, and OR-A conceived and designed the re-analysis of single cell data. AV-J did the computational analysis of single-cell data. UA-PDL and MM-G analyzed single-cell data using progeny and dorothea. AV-J and EM-O performed the enrichment analysis, and the progeny and dorothea analyses. Finally, OR-A applied machine-learning on single cell data. All authors analyzed and interpreted the data. OR-A supervised the research. All authors wrote, discussed, and reviewed the manuscript. All authors contributed to the article and approved the submitted version.

## Conflict of Interest

The authors declare that the research was conducted in the absence of any commercial or financial relationships that could be construed as a potential conflict of interest.

## Publisher’s Note

All claims expressed in this article are solely those of the authors and do not necessarily represent those of their affiliated organizations, or those of the publisher, the editors and the reviewers. Any product that may be evaluated in this article, or claim that may be made by its manufacturer, is not guaranteed or endorsed by the publisher.
