# Supplementary material for: On Deep Landscape Exploration of COVID-19 Patients Cells and Severity Markers
Source: Front Immunol. 2021 Sep 16;12:705646. doi: 10.3389/fimmu.2021.705646 (PMC8481922; doi:10.3389/fimmu.2021.705646)
Supplement: Supplementary file 1 [file DataSheet_1.docx]

On deep landscape exploration of COVID-19 patients cells and severity markers

**Aarón Vázquez-Jiménez^1^, Ugo Ávila-Ponce de León^1,2^, Meztli Matadamas-Guzmán^1,3^, Erick Andrés Muciño-Olmos^1,3^, Yoscelina E. Martínez-López^1,4^, Thelma Escobedo-Tapia^1,5^ & Osbaldo Resendis-Antonio^1,6^ ***

**SUPPLEMENTARY MATERIAL.**


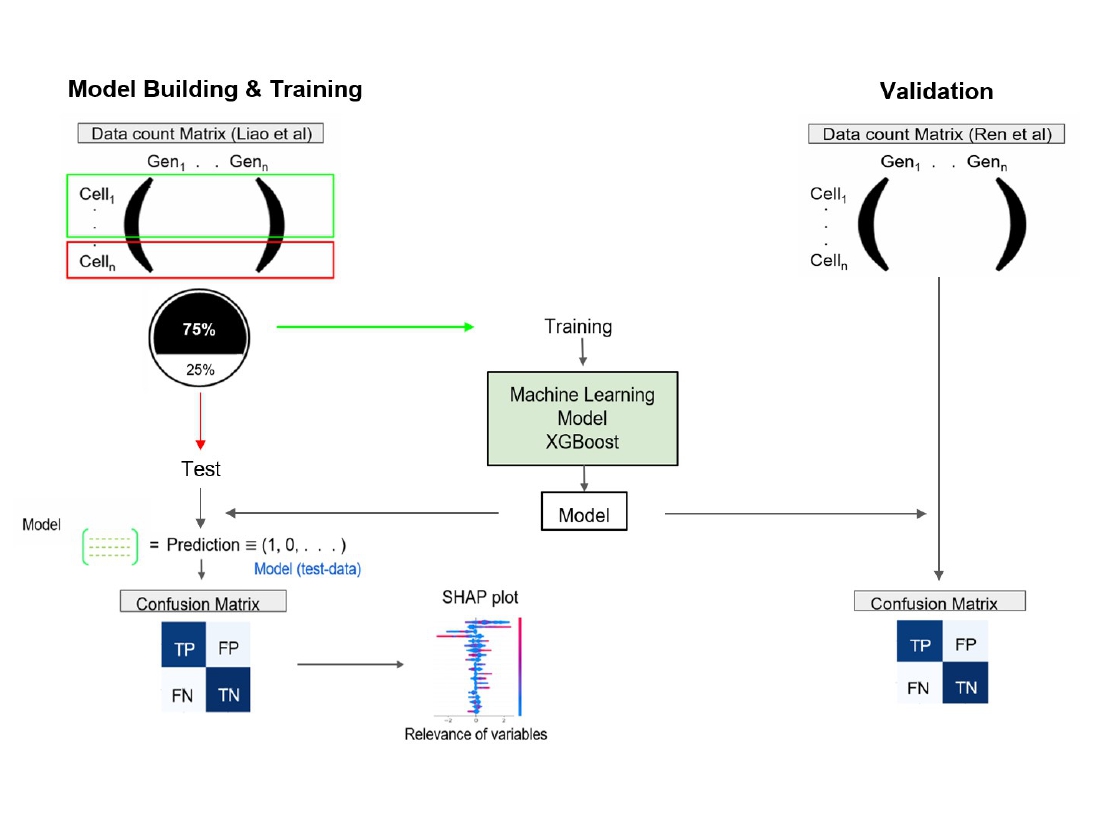


**Figure S1.** Graphical description of Machine Learning analysis. The machine learning model was fed with the count matrix containing cells in rows and gene expressions in columns. Subsequently, 75% and 25% of the complete data from the count matrix were randomly selected as training and test data respectively. The XGBoost model was built with the train data set and the prediction was realized labelling the cells from the count matrices of moderate or severe patients as 0 and 1 respectively. Then, to assess the model performance, or how well the model classifies cells from moderate or severe patients in test data, we calculated the confusion matrix. Subsequently, we evaluated the relevance assessment of characterized genes through the shap-value. Finally, To verify the performance of the XGBoost model in classifying cells in another dataset, we used a completely new dataset.


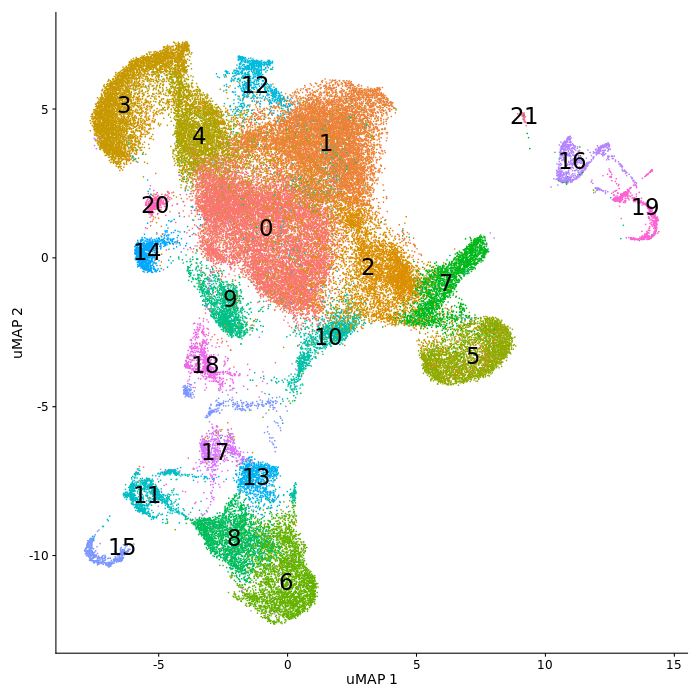


**Figure S2**. Bidimensional uMAP projection for 9 patients (3 moderate and 6 severe) and 3 healthy controls. Data were clustered according to a graph-based approach using seurat pipeline, 22 clusters were found represented with different colors.


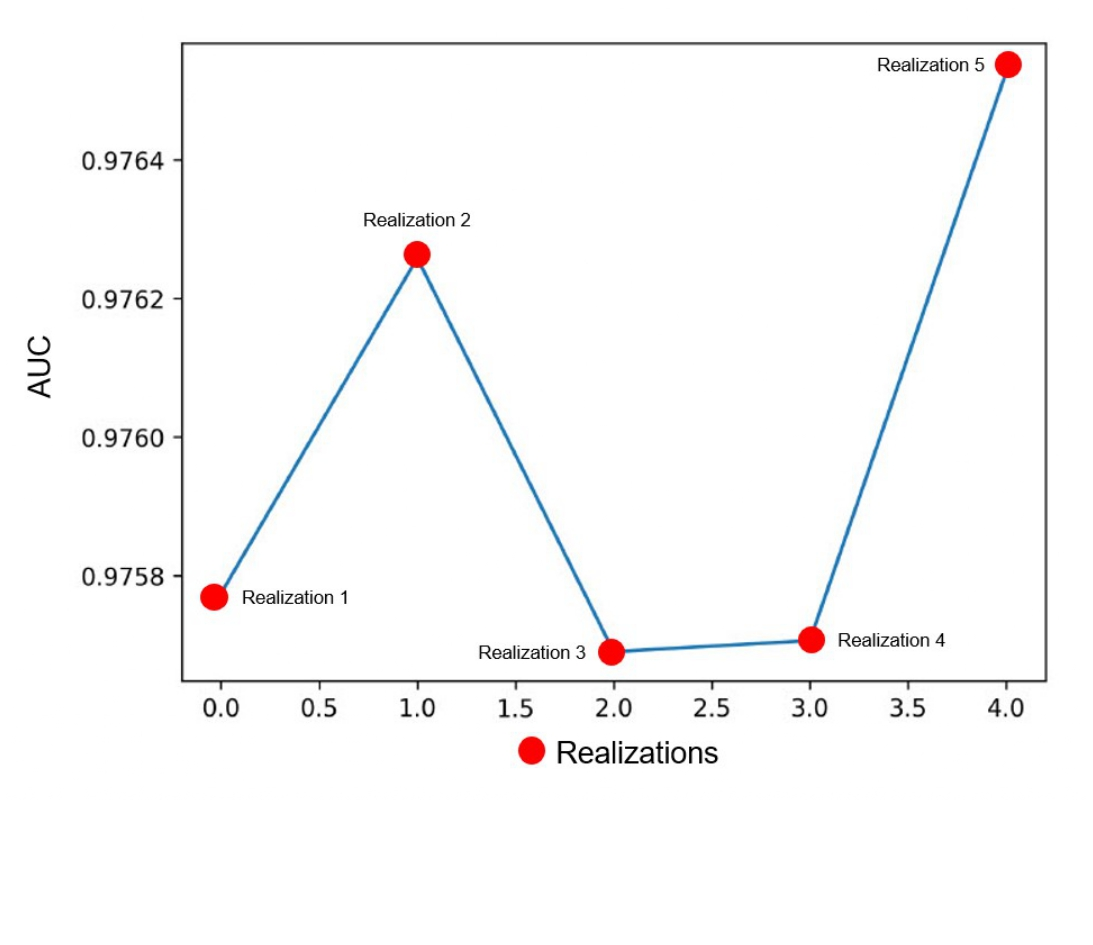


**Figure S3.** Area under the curve (AUC) of ROC curves for the five realizations. On each realization we evaluated the performance of the model and its dependence on the split of the data. We noted that the AUC was on average 0.97 for all the realizations.


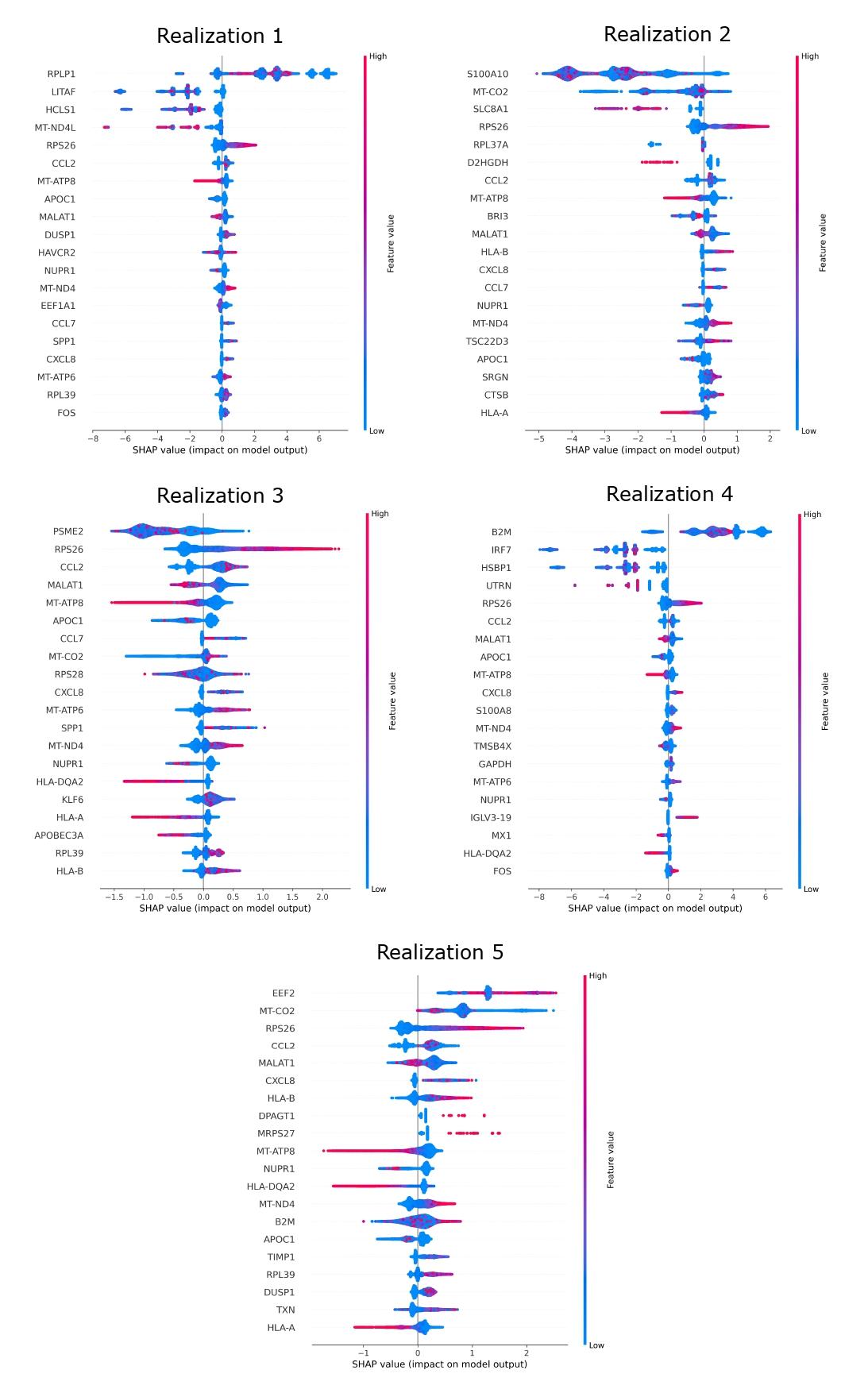


**Figure S4.** Shap plots for the five realizations for the cross-validation test. To evaluate the dependence of the machine learning model of how we split the model into training and testing samples, we applied cross-validation methods with k-split components (k=5). Figure below shows the five realizations obtained from the resampling method applied to the subset of data.

**
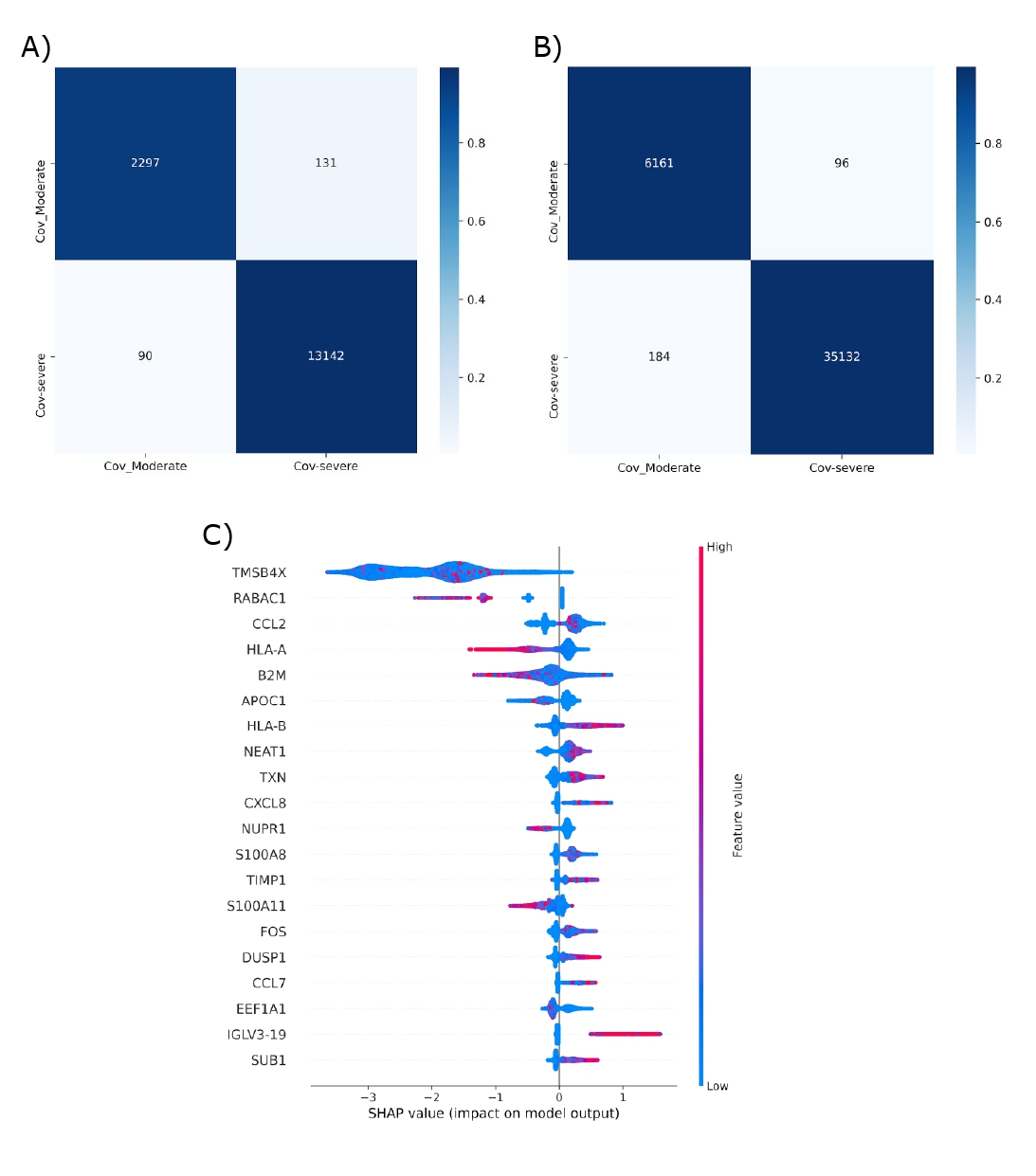
**

**Figure S5.** Confusion matrix for the machine learning model trained with a reduced set of genes. **(A)** Confusion matrix obtained for the test geneset. **(B)** Confusion matrix for an entire new set of scRNAseq data of BALF cells obtained for severe and moderate patients. **(C)** Shap plots obtained for one realization of the model.

**
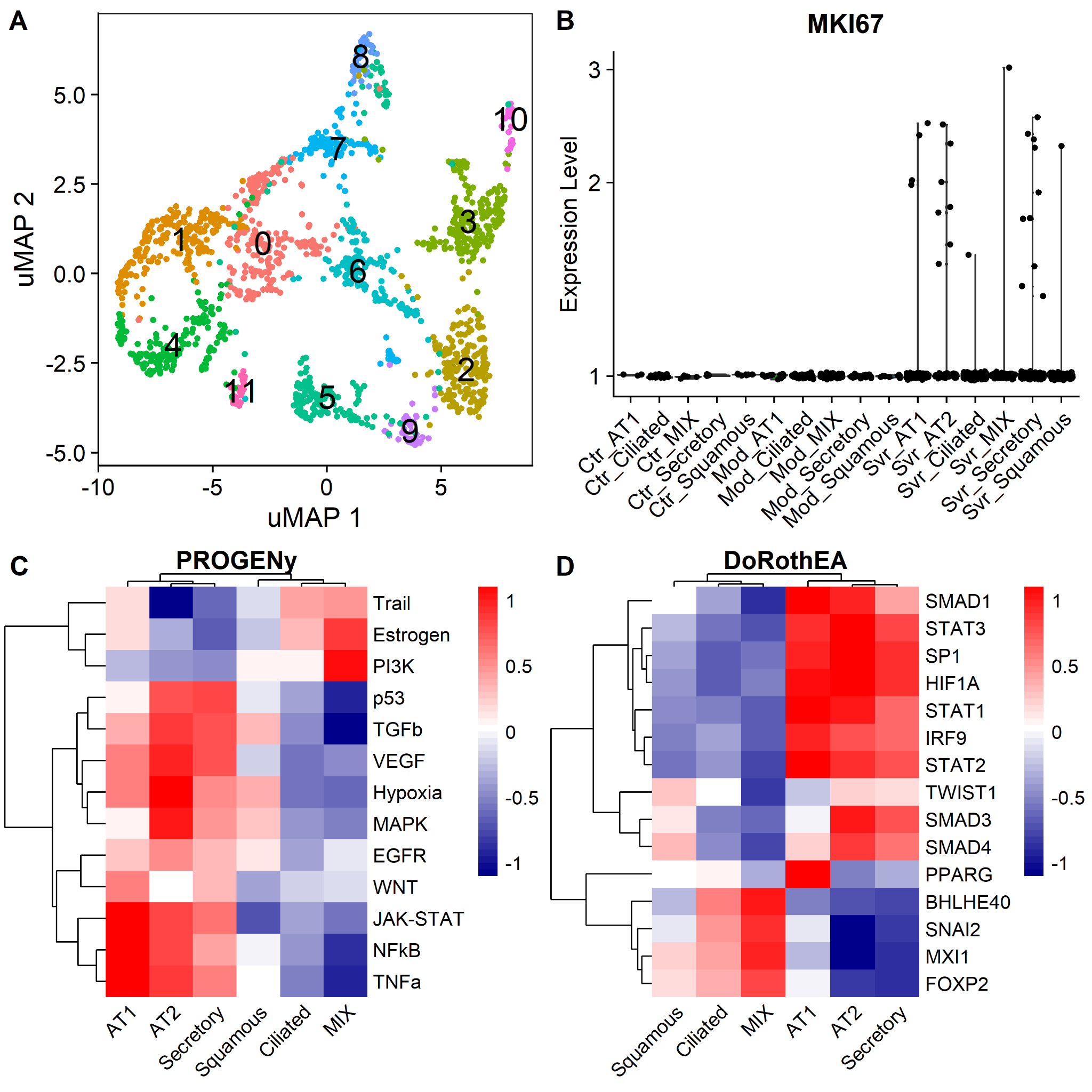
**

**Figure S6.** Epithelial cells diversity analysis. **(A)** Bidimensional uMAP projection for epithelial data from 9 patients (3 moderate and 6 severe) and 3 healthy controls. Data were clustered according to a graph-based approach using seurat pipeline, 12 clusters were found represented with different colors. **(B)** Proliferation marker MKI67 expression for the different epithelial cells subtypes considering the health status. Ctr, Mod, and Svr stand for healthy control, moderate and severe patients, respectively. **(C)** Activation/inactivation pathways analysis for the epithelial subtypes. **(D)** Activation/inactivation analysis for the TFs discussed on the whole-epithelial analysis (TFs denoted in purple along with Figure 3B). Colorbar is related to activation (red) and inactivation (blue) values.

**
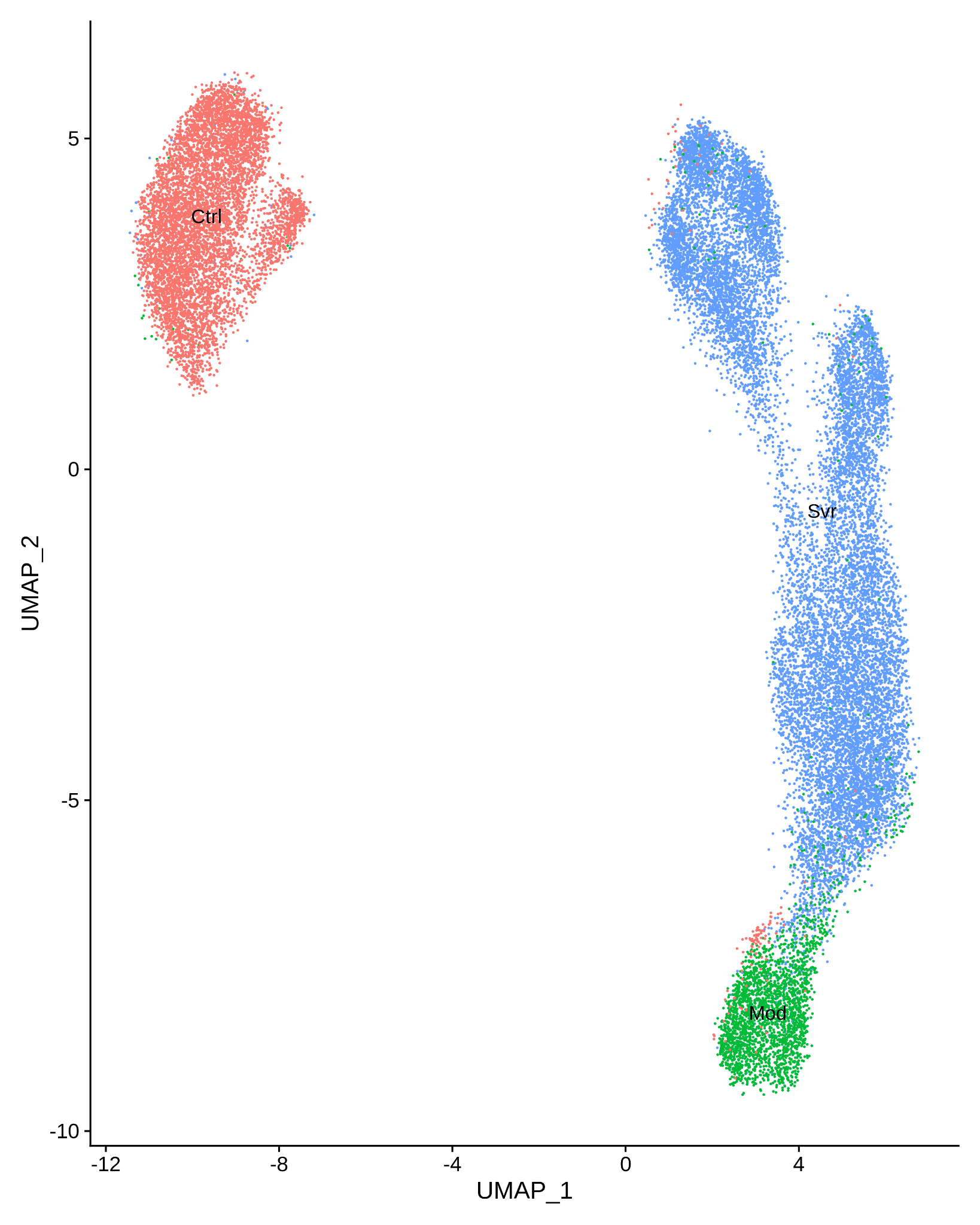
**

**Figure S7.** Macrophage uMAP showing cells on DoRothEA space, colored by disease severity. Crt, Mod and Svr stand for healthy control, moderate and severe COVID-19 patients, respectively.
